# Advancements in textile techniques for cardiovascular tissue replacement and repair

**DOI:** 10.1063/5.0231856

**Published:** 2024-10-17

**Authors:** Abiola Bakare, Hemanth Ponnambalath Mohanadas, Nick Tucker, Waqar Ahmed, A. Manikandan, Ahmad Athif Mohd Faudzi, Shahrol Mohamaddan, Saravana Kumar Jaganathan

**Affiliations:** 1 School of Engineering, College of Health and Science, Brayford Pool, Lincoln LN6 7TS, United Kingdom; 2 Abbott Diabetes Care, Alameda, California 94502, USA; 3 School of Mathematics and Physics, College of Health and Science, Brayford Pool, Lincoln LN6 7TS, United Kingdom; 4 Department of Chemistry, Karpagam Academy of Higher Education, Coimbatore, Tamil Nadu 641021, India; 5 School of Electrical Engineering, Faculty of Engineering, Universiti Teknologi Malaysia, Johor Bahru, Malaysia; 6 Innovative Global Program College of Engineering, Shibaura Institute of Technology, Saitama, Japan; 7 Institute of Research and Development, Duy Tan University, Da Nang, Vietnam; 8 School of Engineering & Technology, Duy Tan University, Da Nang, Vietnam; 9 Biomaterials and Tissue Engineering, School of Engineering and Physical Sciences, College of Health and Science, Brayford Pool, Lincoln LN67TS, United Kingdom

## Abstract

In cardiovascular therapeutics, procedures such as heart transplants and coronary artery bypass graft are pivotal. However, an acute shortage of organ donors increases waiting times of patients, which is reflected in negative effects on the outcome for the patient. Post-procedural complications such as thrombotic events and atherosclerotic developments may also have grave clinical implications. To address these challenges, tissue engineering is emerging as a solution, using textile technologies to synthesize biomimetic scaffolds resembling natural tissues. This comprehensive analysis explains methodologies including electrospinning, electrostatic flocking, and advanced textile techniques developed from weaving, knitting, and braiding. These techniques are evaluated in the context of fabricating cardiac patches, vascular graft constructs, stent designs, and state-of-the-art wearable sensors. We also closely examine the interaction of distinct process parameters with the biomechanical and morphological attributes of the resultant scaffolds. The research concludes by combining current findings and recommendations for subsequent investigation.

## I. INTRODUCTION

In the UK, cardiovascular disease (CVD) accounts for approximately 25% of mortality, or in numerical terms, nearly 160 000 fatalities annually, with coronary heart disease (CHD) as the predominant subtype (Bakare *et al.*, 2023). At present, autologous transplants are viewed as the clinical gold standard for addressing coronary artery occlusions (Herrmann *et al.*, 2019). There have recently been significant advancements in the application of synthetic grafts as alternative remedies for compromised vasculature. The UK is an example of an escalating global mortality due to CVD, which clearly mandates urgent intervention.

Conventional therapeutic modalities for cardiovascular anomalies, such as coronary artery bypass grafting (CABG) (Alasnag *et al.*, 2019), frequently encounter impediments such as thrombotic events, which can lead to rapid vessel occlusion. The inherent limitations of this type of intervention are the risk of re-stenosis coupled with the imminent mortality threat stemming from prolonged waiting times due to the lack of donors (Narayan *et al.*, 2018). To ensure a majority of the patient populace procures enduring and efficacious treatment, an innovative approach to cardiovascular tissue restoration is imperative.

**FIG. 1.**
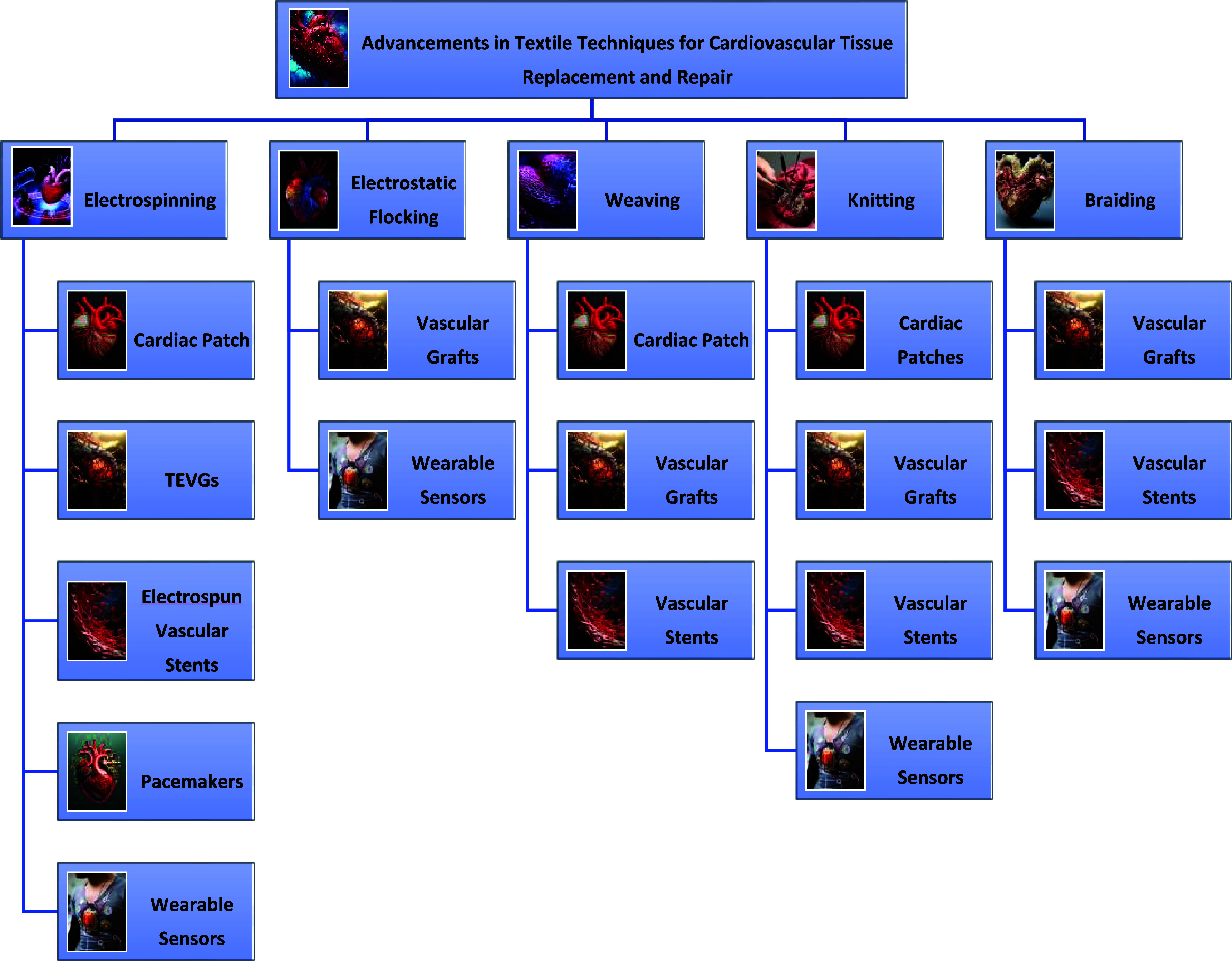
Flow chart of textile fabrication techniques.

In response, tissue engineering professionals have proposed methodologies consisting of the synthesis of biocompatible and biodegradable artificial tissues that mirror the physiology of their biological counterparts (Bambole and Vir Yakhmi, 2016). A variety of fiber technologies have been employed to devise textile-centric scaffolds, leveraging cell-integrated fibers. Figure 1 illustrates a graphical flow chart of these textile fabrication techniques, including electrospinning, weaving, knitting, braiding, and electrostatic flocking.

Graphical flow chart representation of the fiber fabrication technology is depicted in Fig. 1 and explained in detail in this research article. As depicted in Fig. 1, the flow chart outlines the processes involved in each textile fabrication technique. Electrospinning involves the creation of nanofibers through the application of a high-voltage electric field to a polymer solution. Weaving interlaces two sets of yarns at right angles to form a fabric, while knitting constructs fabrics by interlooping yarns in a series of connected loops. Braiding intertwines three or more yarns to form a continuous structure, and electrostatic flocking deposits short fibers onto a substrate using an electric field. These techniques are applied in cardiovascular repairs, such as cardiac patches, vascular grafts, stents, and wearable sensors, all of which are studied in detail under each textile technology.

The research will focus explicitly on these textile technologies in cardiovascular therapy, examining the respective merits and limitations of these methods. Although substantial work has been undertaken on the application of electrospinning, weaving, knitting, and braiding, literature on electrostatic flocking remains sparse. In addition, the influence of procedural variables on the biomechanical and morphological characteristics of the resultant scaffolds will be examined.

By critically appraising the integration of textile methodologies within cardiovascular therapeutic applications, this study aims to detail variant textile modalities and pertinent research insights. Additionally, it will scrutinize the potential benefits and constraints of harnessing these technologies for cardiovascular interventions.

## II. CARDIOVASCULAR ANATOMY, PATHOPHYSIOLOGY, AND TREATMENT STRATEGIES

The heart is a robust muscular organ, essential for circulating blood through the vascular network of arteries within the circulatory system (Li, 2004). Due to continuous rhythmic contractions and mechanical stresses, the cardiovascular tissues—especially the heart and arterial walls—must exhibit anisotropic and elastic properties to ensure efficient blood flow and maintain vascular integrity (Hall, 2016). Textile-based scaffolds must mimic these mechanical properties to function effectively within the cardiovascular system.

Cardiovascular tissues, particularly arteries, possess anisotropic mechanical characteristics, meaning the fibers within these tissues are aligned in a specific orientation to handle directional stress (Luketich *et al.*, 2022). This alignment plays a critical role in maintaining the structural integrity of blood vessels, which must withstand varying pressures during each cardiac cycle. Textile-based technologies, such as electrospinning and weaving, can replicate this anisotropic structure, offering a significant advantage in developing therapeutic materials like vascular grafts and cardiac patches. By aligning fibers similarly to those in native cardiovascular tissues, textile scaffolds can effectively mimic the natural biomechanics of the heart and vessels.

Venous blood with low oxygen levels enters the right atrium via the superior and inferior venae cavae, moves to the right ventricle, and is pumped to the lungs for oxygenation. Oxygen-rich blood returns to the left atrium, passes into the left ventricle, and is then circulated throughout the body via the aorta (Hall, 2016). Arteries transport this oxygenated blood to various body regions, and their walls exhibit anisotropic and elastic properties due to the presence of elastic tissue and smooth muscle, which are critical for regulating blood flow and pressure (Avril, 2018). Replicating these structural and mechanical characteristics is essential in designing vascular grafts and cardiac patches using textile technologies. Textile techniques enable the creation of scaffolds that mimic these anisotropic properties, facilitating the development of grafts and patches that integrate seamlessly with the body's native tissues, thereby reducing the risk of complications such as thrombosis and restenosis (Vahabli *et al.*, 2022).

Cardiovascular disease (CVD) encompasses disorders affecting the heart and blood vessels, primarily caused by lipid accumulation within arterial walls—a condition known as atherosclerosis (Warrell *et al.*, 2016). This leads to arterial narrowing and can result in thrombosis, further restricting blood flow and depriving organs of oxygen and nutrients (Factor *et al.*, 2006). The complexity of cardiovascular tissue structure and function presents challenges in developing effective treatments, highlighting the need for advanced materials that can integrate seamlessly with native tissues (Warrell *et al.*, 2016). Textile scaffolds can address these challenges by providing customizable architectures that promote tissue regeneration and restore normal function.

CVD is the leading cause of mortality worldwide, responsible for 17.9 × 10^6^ deaths in 2016, representing 31% of all global deaths (Mohanadas *et al.*, 2023). Coronary artery disease (CAD), the most prevalent form of CVD, occurs when coronary arteries are occluded by fatty deposits, leading to symptoms such as angina and shortness of breath (Agrawal *et al.*, 2020). Lifestyle changes can help manage CAD, but many patients require medical or surgical interventions to restore arterial blood flow (King *et al.*, 2022).

Traditional treatments for CAD include coronary angioplasty, coronary artery bypass grafting (CABG), and heart transplantation (Jentzer *et al.*, 2014). In angioplasty, a balloon catheter dilates the narrowed artery, followed by stent placement to keep it open (Kapadia and Lau, 2012). CABG uses autologous grafts to bypass blocked coronary segments but has limitations such as graft failure and donor-site complications (Haller *et al.*, 2015; Tara *et al.*, 2015). Heart transplantation is reserved for severe cases but faces challenges like immune rejection and donor shortages (Tong and Khush, 2021). These limitations are partly due to the inability of traditional materials to replicate the mechanical and biological properties of cardiovascular tissues, leading to complications such as thrombosis and restenosis.

For example, textile technologies such as knitting, braiding, and electrospinning allow for the fabrication of vascular stents and grafts that replicate the anisotropic properties of arteries, providing superior strength and flexibility (Vahabli *et al.*, 2022). Additionally, these scaffolds can promote tissue regeneration, offering long-term integration with native tissues and reducing the risk of restenosis or graft failure.

To overcome these limitations, tissue engineering offers innovative solutions by creating biocompatible scaffolds that promote tissue regeneration without adverse immune responses (Balaji *et al.*, 2015). Textile technologies play a significant role in fabricating these scaffolds, as they allow for precise control over the scaffold's mechanical properties, porosity, and architecture, which are crucial for matching the anisotropic and dynamic nature of cardiovascular tissues (Jiang *et al.*, 2021). Techniques such as electrospinning, weaving, knitting, and braiding enable the creation of scaffolds that mimic the hierarchical structure of native tissues, enhancing cellular integration and function. By focusing on the alignment and anisotropy of fibers, textile techniques can create therapeutic materials that better mimic the natural properties of cardiovascular tissues, significantly enhancing performance and longevity in clinical applications (Doersam *et al.*, 2022).

Our study examines these textile-based innovations with potential in cardiovascular device development, highlighting their transformative impact on therapeutic strategies. By focusing on the application of textile technologies in CAD treatment, we aim to showcase how these methods can address the challenges associated with conventional therapies and improve patient outcomes.

## III. LITERATURE REVIEW METHODOLOGY

To conduct a comprehensive research of advancements in textile techniques for cardiovascular tissue replacement and repair, we employed a systematic literature search strategy. This strategy focused on identifying and analyzing relevant scientific literature accessible through databases such as Google Scholar and PubMed. The objective was to gather insights into the application of various textile technologies in cardiovascular methodologies, including the fabrication of cardiac patches, vascular graft constructs, stent designs, and wearable sensors.

The search criteria were divided into three primary sections:

1. Keywords related to textile technology: We used terms such as “electrospinning,” “electrostatic flock*,” “weav*,” “knit*,” and “braid*” to capture the range of textile techniques applied in biomedical engineering.
2. Terminology for cardiovascular applications: This included specific terms like “cardiac patch,” “vascular graft,” “vascular stents,” “pacemaker,” “heart valves,” “valved blood vessels,” “occludes,” and “wearable sensors” to ensure the search was relevant to cardiovascular tissue engineering and therapeutic devices.
3. Application-specific terminology: Keywords such as “biomimetic scaffolds,” “biocompatibility,” “mechanical properties,” “cell proliferation,” and “tissue engineering” were used to identify studies focusing on the functional and structural integration of textile-based constructs in cardiovascular therapies.

To enhance the rigor of our research, we implemented specific inclusion and exclusion criteria. Inclusion criteria included (1) peer-reviewed articles published in English between January 2000 and October 2023; (2) studies focusing on the application of textile technologies in cardiovascular tissue engineering and repair; (3) research presenting original experimental data, including *in vitro*, *in vivo*, or clinical studies; and (4) articles discussing mechanical properties, biocompatibility, or cellular interactions of textile-based scaffolds. Exclusion criteria involved (1) articles not directly related to textile fabrication techniques; (2) studies without accessible full texts; and (3) publications focusing on non-cardiovascular applications of textile technologies.

Data extraction involved systematically collecting information on study objectives, textile fabrication techniques employed, materials used, experimental designs, key findings, and conclusions. We also noted details regarding control setups, statistical methods employed, and assessments of reproducibility to evaluate the quality and reliability of the studies. Data synthesis involved categorizing the studies based on textile techniques and cardiovascular application. We analyzed the mechanical properties, biocompatibility, and cellular responses reported, comparing them across different fabrication methods. Statistical analyses presented in the studies were studied to assess the significance of the findings.

By integrating these data, we aim to provide a comprehensive overview of how textile technologies are advancing cardiovascular therapies, highlighting both the potentials and limitations observed in the literature.

## IV. ELECTROSPINNING: PRECISION IN NANOFIBER SCAFFOLD FABRICATION

Electrospinning is an advanced textile method tailored for scaffold production, encompassing the generation of ultrafine fibers by applying a high-voltage electric field to a polymer solution or melt, which is then ejected through a spinneret (Jiang *et al.*, 2021). This technique has been heralded for its ability to produce continuous nanofibers that are homogeneous in diameter and composed of a variety of materials, a significant advancement in textile technology. Electrospinning produces nanofibrous scaffolds with fiber diameters ranging from a few nanometers to micrometers thick (Mani *et al.*, 2024). The inherent capability of electrospinning allows meticulous control over polymer fiber deposition onto designated substrates, thus enabling the formulation of intricate three-dimensional (3D) nanofiber structures.

**FIG. 2.**
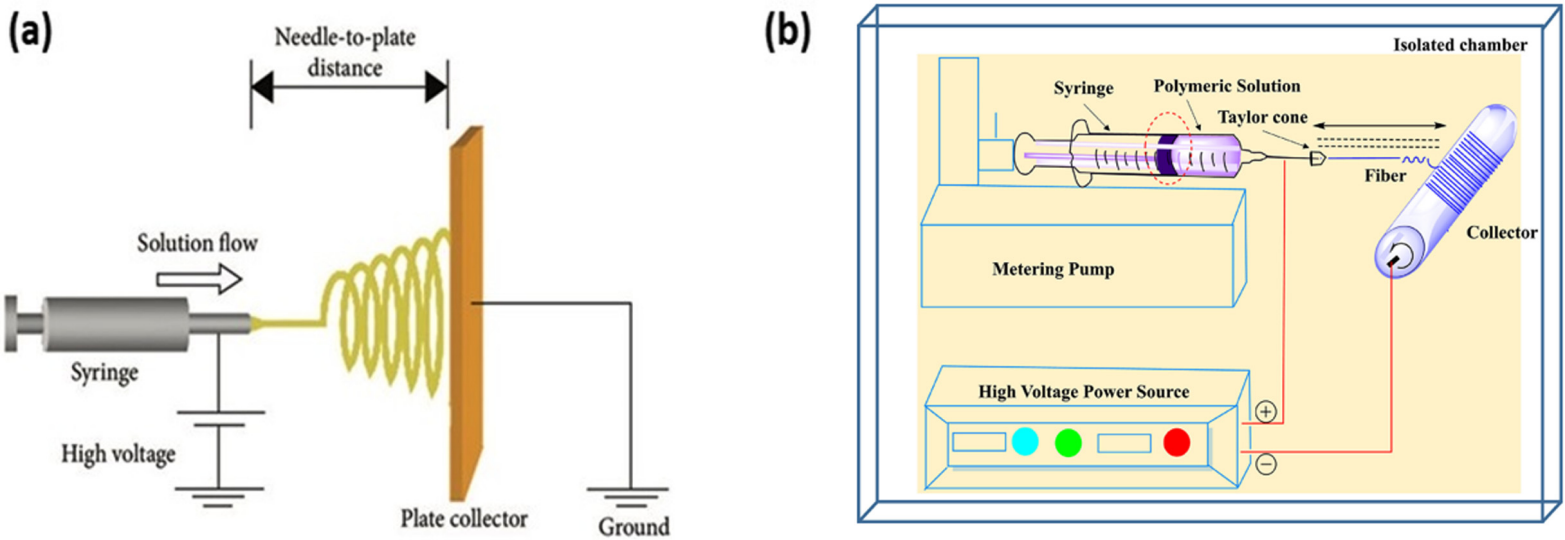
(a) Setup of electrospinning of 2D scaffold [Reproduced with permission from Zhou *et al.*, Int. J. Biomater. **1**, 953636 (2018). Copyright 2018 Authors, licensed under a Creative Commons Attribution (CC BY) License]. (b) Setup of electrospinning of 3D scaffold for fabrication of LP nanofibers. Reproduced with permission from Garkal *et al.*, OpenNano **8**, 100098 (2022). Copyright 2022 Authors, licensed under a Creative Commons Attribution (CC BY) License (Garkal *et al.*, 2022).

The fundamental mechanism behind electrospinning involves a high-voltage power source, a spinneret, and a conductive collector as shown in Fig. 2(a) (Zhou *et al.*, 2018). Figure 2 shows the setup for both the electrospinning setup for 2D and 3D scaffold. As the polymer solution is expelled from the spinneret, surface tension is overcome by internal electrostatic repulsion due to the applied high voltage, leading to fiber being emitted from the spinneret and ultimately collected on a grounded collector. The evolving electrostatic forces result in the formation of a conical structure known as the Taylor cone on the surface of the droplet emitting from the spinneret. As this cone surface charge intensifies, a polymer jet emerges, which if the surface charge is high enough subsequently bifurcates into multiple fiber strands. At lower field strengths, a single fiber is formed. The jet changes to a fiber as the solvent evaporates (Wang *et al.*, 2019).

The properties and end morphology of these nanofibers are determined by the following factors: attributes of the polymer solution, including parameters such as molecular weight (usually perceived as viscosity); the variables inherent to the electrospinning process, such as applied voltage and feed rate; and the ambient conditions, such as temperature and humidity (Shi *et al.*, 2022). Furthermore, the molecular weight of a polymer has a profound impact on characteristics like its melting point, solubility, and mechanical strength.

A broad range of materials, from collagen and silk to polyurethanes, can be employed within the electrospun, allowing the creation of structures tailored for diverse applications (Zhou *et al.*, 2015). By blending or creating core/shell structures (known as co-axial spinning) with these polymers, the resultant scaffolds can be optimized in terms of biocompatibility, conductivity, and mechanical resilience (Bertuoli *et al.*, 2019).

Electrospun fibers can be collected, either randomly using a stationary plate or in a semi-aligned manner with a rotating mandrel, offering versatility in the orientation and mechanical properties of the scaffold (Wang *et al.*, 2022). Nanofibers are used in various domains, including tissue engineering, bio-textiles, and battery research. Their significant potential in regenerative medicine, drug encapsulation, and commercial avenues is indicated by the growing adoption and evolution of electrospinning technologies over the past decade (Arik, 2024). In the biomedical realm, these scaffolds play a pivotal role in emulating the extracellular matrix, thus fostering cellular adherence, viability, and tissue regeneration. Their application extends to realms such as cardiac patches, vascular grafts, and even wearable sensors, illustrating the expansive potential of electrospinning in healthcare and beyond (Ramasubramanian *et al.*, 2022).

### A. Cardiac patch

Cardiac patches are advanced biomimetic scaffolds designed for the restoration and repair of compromised myocardium, amalgamating progenitor cells with the potential to differentiate into cardiomyocytes and precisely tailored scaffold matrices (Zhang *et al.*, 2022). These patches, when temporarily affixed to an infarcted ventricular surface, have demonstrated superior efficacy in restoring the functionality of injured cardiac tissues (Yao *et al.*, 2022). Fundamental to the functionality of these patches are their dual capacities: offering essential mechanical reinforcement and orchestrating targeted cellular or biomolecular therapeutic delivery, hence stimulating regenerative pathways (Wang *et al.*, 2022).

Electrospinning has emerged as a useful technique for the fabrication of cardiac patches with enhanced physicochemical and biological attributes (Cristallini *et al.*, 2020). For instance, a cardiac patch was synthesized using electrospun collagen fibers, specifically atelocollagen along with acidic and basic fibrous collagen (Kitsara *et al.*, 2015). Their *in vivo* trials used a murine model of dilated cardiomyopathy and revealed superior performance of electrospun fibers derived from atelocollagen in comparison to alternatives. Notably, the biocompatibility of this scaffold was pronouncedly commendable, with negligible inflammation post-implantation over a fortnight.

The adaptability of the electrospinning technique can be credited to its ability to incorporate a plethora of polymers, each characterized by distinct physicochemical attributes tailored to specific requirements (Ghosh *et al.*, 2023). Moreover, this method facilitates the incorporation of cells within the electrospun solutions, engendering cell-laden matrices. The viability, phenotypic characteristics, and growth factor expression in electrospun patches of varied thicknesses were investigated, utilizing both bone marrow and human cardiac stem cells (Chen and Kan, 2018). Electrospun using a 13% poly-caprolactone (PCL) solution in an 80:20 v/v ratio mixture of dichloromethane/dimethylformamide (DCM/DMF), the results indicated remarkable cellular survivability within these PCL patches, even in the more internalized regions. Their subsequent transplantation into a rodent myocardial infarction model demonstrated graft compliance and impressive survivability rates, thus underscoring the potential efficacy of PCL-based cellular patches for cellular transplantation.

For cardiac tissue regeneration, electrospun nanofiber scaffolds have become a focal point of research (Zhang *et al.*, 2022). Conceived to remediate the injured myocardium, these scaffolds proffer both mechanical and regenerative support upon affixation to the cardiac surface. Incorporating methodologies such as electrospinning and 3D bioprinting, these cardiac patches emulate the native extracellular matrix, thereby providing essential mechanical support to resident cells (Yong *et al.*, 2020). Beyond this, alternative regenerative modalities include cell sheet layering, advanced 3D bioprinting, hydrogel matrices, and decellularized extracellular matrices (Wang *et al.*, 2022). Such patches can be imbued with diverse cell phenotypes, encompassing human mesenchymal stem cells (hMSCs), endothelial cells (ECs), and human cardiomyocytes (hCMs), alongside vital growth factors and therapeutic agents that augment cellular functionality. The strategic assembly of these fibers allows architectural mimicry of the native cardiac tissue, underscoring the potential of this technique (Zhe *et al.*, 2023). The versatility of electrospinning extends to other biomedical paradigms, such as vascular grafts, vascular stents, pacemakers and wearable technology, which will be explained in Secs. IV B–IV E.

### B. Tissue-engineered vascular grafts (TEVGs)

**FIG. 3.**
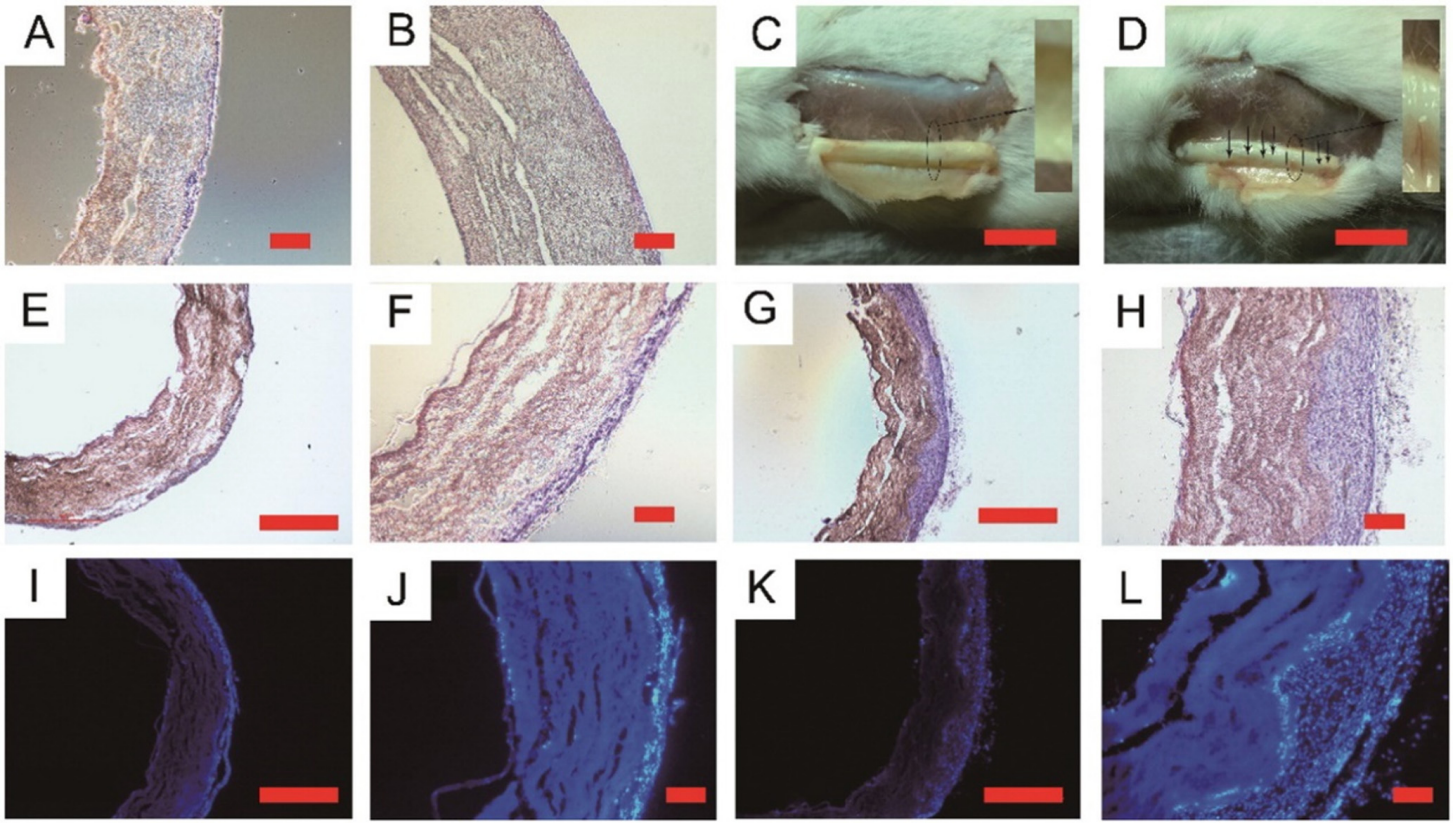
The image features stereomicroscopic views of explanted vascular grafts fabricated using a co-electrospinning process to create composite vascular grafts comprising of PCL/GT (poly-caprolactone/gelatin) and PVA (polyvinyl alcohol) polymers. It includes cross sections stained with H&E and DAPI at different intervals post-implantation. The first set (A-B) shows type A (PCL/gelatin) and type B (PCL/gelatin/PVA) scaffolds at 3 weeks post-implantation. C-D The same scaffolds after 8 weeks. Additional cross sections (E-H for H&E, I-L for DAPI staining) at 8 weeks illustrate the structural differences between type A and type B scaffolds. Scale bars vary, indicating the size differences in the images. Reproduced with permission from Tan *et al.*, Mater. Sci. Eng. C **67**, 369–377 (2016); licensed under a Creative Commons Attribution (CC BY) License.

Tissue-engineered vascular grafts (TEVGs) have emerged as advanced alternatives to conventional vascular grafts traditionally employed in therapeutic regimes. At its core, a TEVG comprises a meticulously fabricated scaffold, judiciously seeded cells, and the requisite environmental stimuli, encompassing growth factors and mechanical cues, to instigate tissue morphogenesis (Gupta and Mandal, 2021). It is imperative that post-implantation, a TEVG seamlessly integrates with contiguous vasculature, withstands hemodynamic forces, and ensures non-leaky perfusion. Two quintessential attributes of TEVGs are biocompatibility and bioactivity. Tan *et al.* (2016) developed composite vascular grafts using PCL/GT and PVA polymers, those stereomicroscopic views are shown in Fig. 3, through co-electrospinning, with a key enhancement of heparin immobilization (Tan *et al.*, 2016). These grafts displayed promising mechanical properties suitable for arterial replacement, significant porosity aiding cell growth, and strong anti-thrombogenic traits, showcasing their potential in vascular tissue regeneration. Moreover, the mechanical properties, ligand adhesive capabilities, growth factor presentation, and the kinetics of transport and degradation of the scaffold materials should emulate the pertinent extracellular matrix (ECM) milieu to an optimal degree (Ahadian *et al*., 2017). While an exact simulacrum of the indigenous tissue architecture is not mandatory, the TEVG, as a functional construct, must satisfy certain specific criteria. For instance, a burst strength exceeding 26 kPa is essential to obviate rupture under fluctuating blood pressure (Gong and Niklason, 2006). Furthermore, congruence with the adjacent host vessel and an anti-thrombogenic lumen (via autologous endothelium) is paramount (Stegemann *et al.*, 2007). A scaffold's ability to bestow initial mechanical stability is pivotal, with the anticipation that native vessel attributes will be progressively assimilated through remodeling, repair, and growth post-implantation. Additionally, implanted TEVGs should curtail intimal hyperplasia and foster arterial tissue regeneration (Vahabli *et al.*, 2022).

Recent experimental endeavors have significantly advanced our understanding of vascular grafts. A tubular hyaluronic acid/collagen nanofibrous scaffold was developed for vascular rejuvenation in a rabbit model. After replacing the carotid artery in 6-week-old rabbits, it was observed that the endothelialized hyaluronic acid/collagen nanofibrous composite scaffold grafts effectively maintained vascular patency. The scaffold exhibited mechanical robustness comparable to the native artery upon retrieval, demonstrating sufficient structural integrity (Niu *et al.*, 2021). Similarly, the *in vivo* potential of an electrospun PCL/fibrin vascular graft was explored. By combining PCL and fibrin with 98% formic acid (in a weight ratio of 20:80), a nanofibrous graft was created that showed impressive mechanical strength, enhanced compliance, and superior degradation kinetics (Zhao *et al.*, 2021). Remarkably, post-implantation into a rat abdominal aorta, the graft facilitated neoartery formation and accelerated endothelialization over 9 months. Within 3 months, the PCL/fibrin vascular graft displayed increased micro-vessel density and reduced calcification zones, promoting cellular infiltration and proliferation. The electrospun PCL/fibrin tubular vascular graft shows promise as an artificial blood artery scaffold with extended *in vivo* functionality.

### C. Electrospun vascular stents

A vascular stent, in its essence, is a very small tube introduced into a compromised artery, acting as a structural support to restore and maintain luminal patency. Beyond its role of ensuring unhindered blood flow, modern stents are tasked with more nuanced functionalities. They should exhibit anticoagulant and antithrombotic properties to avert potential complications such as stenosis and occlusion (Badv *et al.*, 2020). In light of this, more researchers are employing electrospinning technology, innovating with diverse materials to concurrently promote vascular intimal endothelial cell proliferation and inhibit excessive smooth muscle cell (SMC) growth.

Electrostatically spun tubes, on the other hand, serve as vascular scaffolds by providing a three-dimensional structure that not only maintains vessel patency but also actively supports the regeneration and integration of new tissue (Tolba, 2020). Unlike vascular grafts, which replace or bypass vessels, electrospun scaffolds are designed to promote cellular adhesion and proliferation, facilitating the growth of new endothelial and smooth muscle cells (Rickel *et al.*, 2021). This makes them an essential tool in regenerative medicine, where the goal is to restore native vascular functions rather than merely replace the damaged vessel.

To further the objectives of vascular tissue regeneration, bioactive agents such as heparin (Kuang *et al.*, 2020), growth factors (Han *et al.*, 2013), and other specialized chemicals (Strobel *et al.*, 2018) are typically integrated onto the surface of electrospun tubular membranes. The efficacy of these technologically advanced stents has been rigorously appraised using animal models, including rat abdominal aortas and rabbit carotid arteries, as demonstrated by various studies (Wu *et al.*, 2018). These scaffolds can act as temporary supports, gradually biodegrading as the native tissue regenerates, allowing the body to heal itself while providing mechanical stability during the healing process.

While vascular stents primarily serve to maintain vessel patency by propping open narrowed arteries, vascular grafts are used to replace or bypass diseased or damaged blood vessels (Gupta and Mandal, 2021). Vascular scaffolds, distinct from grafts, support tissue regeneration and are often used in tissue engineering to promote the formation of new blood vessels rather than merely serving as a conduit for blood flow. Vascular scaffolds, on the other hand, are three-dimensional structures that support cell growth and tissue regeneration, often used in tissue engineering to promote the formation of new blood vessels (Liu *et al.*, 2020).

A tri-layered stent was constructed using electrospinning and a poly(l-lactide-co-caprolactone) membrane (Liu *et al.*, 2020). This innovative design included an external layer with a structured texture, ideal for smooth muscle cell (SMC) proliferation and mechanical integrity, while the internal layer was densely crafted to support endothelial cell growth. The intermediary layer was tailored to facilitate SMC proliferation, resulting in a stent with superior tensile strength.

Recent advancements in bi-layer scaffold design have shown promising results for clinically relevant tissue-engineered vascular substitutes. A notable example is a bilayer vascular scaffold designed to promote rapid endothelialization *in situ*, along with the alignment and infiltration of SMCs (Wang *et al.*, 2021). The inner layer of this scaffold, composed of electrospun poly(l-lactide-co-caprolactone) and collagen (PLCL/COL) nanofibers, was infused with heparin and vascular endothelial growth factors (VEGF). The outer layer consisted of circumferentially aligned PLCL/COL nanofiber yarns embedded with platelet-derived growth factors (PDGFs), with concentrations increasing from the outermost to the innermost regions. This scaffold was fabricated using a combination of coaxial electrospinning for the inner layer and a modified process integrating coaxial with conjugated electrospinning for the outer layer, followed by cross-linking with glutaraldehyde vapors. The resulting vascular scaffold displayed compliance comparable to that of a human saphenous vein and superior to commercial polytetrafluoroethylene (PTFE) grafts. *In vitro* studies revealed a sustained release of VEGF at higher rates compared to PDGF over a month. The scaffold demonstrated excellent biological performance, with rapid endothelialization on the luminal surface and oriented smooth muscle cells infiltrating the vascular wall within 2 months post-implantation in a rat abdominal aorta defect model. Additionally, the outermost layer produced collagen-rich connective tissues, indicating the scaffold's potential for *in situ* vascular repair or regeneration. These examples illustrate the critical role of electrospun scaffolds in supporting not just mechanical stability but also biological integration, a key differentiator from vascular grafts. Electrospun scaffolds facilitate the regeneration of native tissues, making them highly suitable for long-term vascular healing and repair.

Another innovative design involved the creation of a small diameter balloon-expandable stent, encapsulated within dual layers of electrospun polyurethane nanofibers (Uthamaraj *et al.*, 2016). The controlled thickness of this nanofibrous membrane was achieved by electrospinning a 15% (m/v) polyurethane solution in dimethylacetamide (DMA) onto a rotating mandrel. When implanted into swine models, these stents demonstrated robust mechanical integrity without occlusion or failure. The stent's flexibility and resilience were evidenced by its ability to crimp and expand without any structural damage. These findings support the premise that electrospinning for medical objectives allows for novel interventions in coronary procedures.

### D. Pacemakers

Artificial pacemakers are sophisticated electronic devices implanted in the thoracic region and are specifically designed to manage certain cardiac arrhythmias that can lead to bradycardia or intermittent heart rhythms (Mulpuru *et al.*, 2017). This management is achieved by imparting periodic electrical impulses to modulate the cardiac rhythm. Artificial pacemakers render therapeutic benefit to numerous patients, significantly enhancing longevity (Weigel *et al.*, 2018). However, the success of these devices critically depends on maintaining an unbroken electrical connection with the myocardium. To ensure this, the pacemaker electrodes are traditionally designed with hooks or screws, which become embedded within post-implantation scar tissue.

However, these metallic electrodes are not devoid of limitations. Potential risks include malfunctioning in certain electromagnetic fields and a chance of untoward reactions or infections due to limited biocompatibility with implant materials, which, if unaddressed, may lead to severe consequences (Liu *et al.*, 2023). An emerging paradigm that directly connects electrospinning technology with cardiovascular repair is the development of three-dimensional, tissue-mimetic electrode scaffolds. These scaffolds, created through electrospinning, offer a promising alternative to traditional planar metal electrodes by mimicking the extracellular matrix, which facilitates improved biocompatibility and tissue integration (Weigel *et al.*, 2018; Azimi *et al.*, 2021).

In a notable study, a conductive, porous fiber scaffold was crafted by electrospinning a polyacrylonitrile (PAN)/*N*,*N*-dimethylformamide (DMF) mixture, followed by high-temperature post-processing (Weigel *et al.*, 2018). This scaffold was populated with cardiomyocytes derived from human induced pluripotent stem cells (hiPSC-CM), leading to the formation of autonomously beating cellular clusters and a significantly enhanced electroactive surface. The electrospun scaffold demonstrated superior regenerative potential, reduced inflammatory responses, and minimal fibrosis, making it a valuable application in pacemaker technology, as it ensures a more seamless integration with myocardial tissue.

Furthermore, the application of a biocompatible piezoelectric polymer-based nanogenerator (PNG) constructed using electrospinning techniques offers an innovative self-sustaining power source for pacemakers. By utilizing poly(vinylidene fluoride) (PVDF) combined with zinc oxide (ZnO) and reduced graphene oxide (rGO), this electrospun PNG could harness energy from cardiac movements, highlighting electrospinning's relevance not only in electrode integration but also in powering cardiovascular devices (Azimi *et al.*, 2021).

### E. Wearable sensors

Increasing interest in wearable sensors for real-time health diagnostics can be attributed to recent technological advances. These sensors have evolved from traditional rigid electronics to flexible, biocompatible devices that can conform to the skin, making them highly relevant for continuous cardiovascular health monitoring (Kulkarni *et al.*, 2024).

Electrospinning technology has played a critical role in the development of piezoelectric pressure sensors, which have emerged as frontrunners in addressing challenges such as power autonomy, flexibility, and adaptability in wearable health devices. These electrospun sensors provide platforms for real-time monitoring of cardiovascular parameters, such as heart rate and blood pressure, with a degree of precision and non-invasiveness that was previously unattainable (Zhang *et al.*, 2022).

For example, smart textile electrospinning has enabled the integration of piezoelectric fibers into wearable fabrics, which can monitor biomechanical movements related to cardiovascular health, such as respiration and pulse. These advancements facilitate early detection of cardiovascular disorders and provide data for guiding therapeutic interventions (Ahmed *et al.*, 2022). Additionally, electrochemical textiles, fabricated using electrospinning, are capable of monitoring sweat metabolites, offering real-time insights into cardiovascular physiology and aiding in disease management (Qiao *et al.*, 2020).

Electrospinning's role in wearable diagnostics extends the technology's application from simple monitoring to more complex, patient-specific therapeutic interventions, ensuring better health outcomes and disease management in cardiovascular care.

### F. Significance and constraints in nanofiber fabrication

Among methods for nanofiber synthesis, electrospinning stands out as an especially versatile technique. This method facilitates the generation of a wide range of materials, encompassing polymers, composites, ceramics, and metals, either directly or through post-spinning modifications. What distinctly differentiates electrospinning from other nanofiber genesis techniques is the ability to produce a wide range of fiber assemblies. Such capabilities augment the functional attributes of nanofiber-centric products, allowing for bespoke modifications tailored to specific application needs (Teo and Ramakrishna, 2006). Through the control of parameters in electrospinning, it is possible to influence scaffold porosity, pore size distribution, and overall architecture. This is achieved by varying the constitution, scale, and orientation of the constituent fibers (Bhattarai *et al.*, 2018).

However, like all technologies, electrospinning is not without its challenges. In the pursuit of fabricating highly efficacious and therapeutic functional engineered cardiac tissues (fECTs), the extant nanofiber scaffold methodologies encounter specific hurdles. Prominent among these are as follows:

• An inherent low porosity impedes the deeper penetration of seed cells into the scaffold matrix.
• The challenges associated with cultivating cardiomyocytes on rigid substrates, which often emulate the biomechanics of post-infarct fibrotic scars.
• The overarching impediments in advancing the current state of the art in nanofiber scaffold technology.

Furthermore, several limitations encumber the broader adoption of electrospinning. Residual solvent toxicity post-fabrication and the comparatively suboptimal mechanical robustness of the resultant scaffolds, especially for applications demanding high mechanical integrity, are noteworthy concerns (Wood *et al.*, 2016).

Beyond the realm of electrospinning, electrostatic flocking emerges as an alternative textile modality, employing high voltage for potential applications in tissue engineering. However, comprehensive investigations into its full potential remain nascent. Section V will examine this technique.

## V. ELECTROSTATIC FLOCKING

**FIG. 4.**
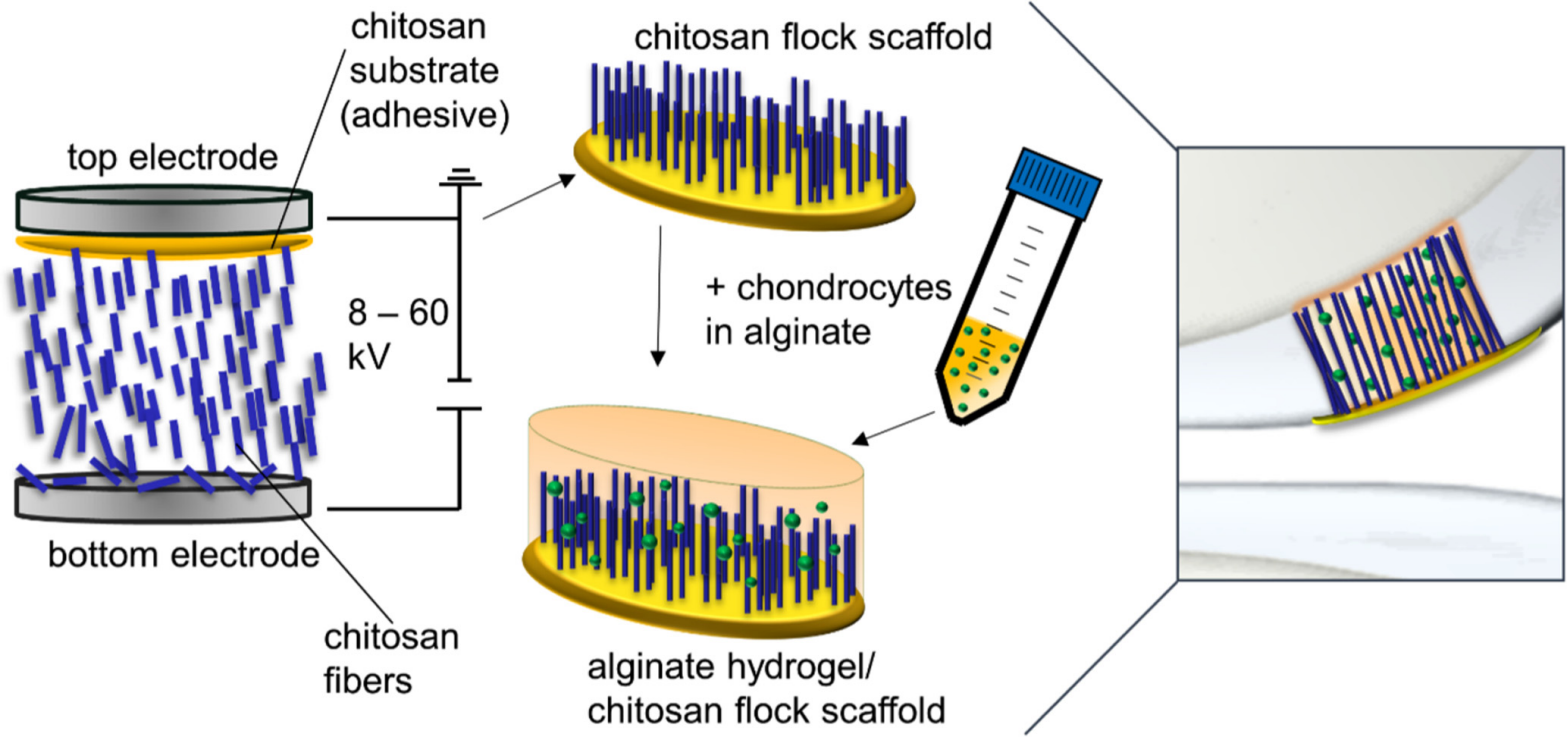
The image depicts electrostatic flocking where chitosan fibers are propelled into a viscous chitosan layer under an electric field. The scaffold is dried, sterilized, and seeded with chondrocytes in alginate. Cells are immobilized through cross-linking, forming a scaffold ready for cartilage defect implantation. Reproduced with permission from Gossla *et al.*, Int. J. Mol. Sci. **22**, 9341 (2021). Copyright 2021 Authors, licensed under a Creative Commons Attribution (CC BY) License.

Electrostatic flocking, a sophisticated and established textile manufacturing technique, harnesses coulombic forces to direct short fibers from a charging source onto an adhesive-laden substrate. The resultant architecture comprises densely packed, aligned fibers oriented perpendicularly to the substrate (McCarthy *et al.*, 2021). The core components of electrostatic flocking include substrates, adhesives, and small-scale fibers (typically ranging from micrometers to millimeters) known as flock fibers. These fibers are preliminarily sifted onto a charging electrode (Gossla *et al.*, 2016). A voltage is incrementally applied until a sufficient charge accumulates on the fiber surfaces. Post charging, these fibers are attracted toward a grounded electrode, where they embed into an adhesive-saturated substrate, thereby giving rise to an assembly reminiscent of an aligned fibrous forest. Within this electrostatic milieu, the fibers align *en route* toward the adhesive-rich substrate, adhering perpendicularly, imparting a velvety texture to the surface. Two salient advantages of this technique are the unparalleled surface-to-volume ratio of the fibers and the subsequent textile formations. Close control over flock fiber diameter is an important parameter influencing both fiber dynamics and orientation (Terakawa *et al.*, 2022). Figure 4 illustrates the process of electrostatic flocking, where chitosan fibers are propelled into a viscous chitosan layer under an electric field (Gossla *et al.*, 2021).

Electrostatic flocking, by virtue of its precision and versatility, is used in many areas. Industries employ it to engineer intricate surfaces on diverse materials, such as paper, plastics, metals, and ceramics (Hitzbleck *et al.*, 2013). Its potential in sculpting porous and anisotropic structures has also been used in tissue engineering (Gossla *et al.*, 2021). However, in the past decade of tissue engineering research, progress has been impeded by challenges. These include stringent electrical conductivity requirements for the fiber, constraints in individual flock fiber production, and a relative dearth of expertise (McCarthy *et al.*, 2021). Consequently, work investigating tissue-engineered constructs via electrostatic flocking has not advanced far. Preliminary work has been centered around vascular grafts and wearable sensors. Yet, noteworthy gaps persist, especially in the areas of electrostatic flocking for cardiac patches, vascular stents, and pacemaker tissue engineering.

### A. Vascular grafts

Electrostatic flocking, although promising as a effective technique for cell cultivation in tissue engineering, has not advanced far in application, especially concerning vascular grafts. A paucity of research has so far limited the use of electrostatic flocking in making fibrous scaffolds tailored for vascular graft applications (Tonndorf *et al.*, 2018).

One pioneering approach involves fabricating vascular grafts by electrostatically flocking poly-caprolactone (PCL) microfibers, incorporating 0.5% silver nanoparticles (AgNP), onto substrates created through a combination of three-dimensional (3D) printing, electrospinning, and thin-film casting (McCarthy *et al.*, 2021). A biocompatible chitosan adhesive was used to attach the fibers to the substrates. These engineered scaffolds, tested via subcutaneous implantation in rat models, demonstrated promising angiogenesis, with the formation of new blood vessels, significant cell infiltration, and favorable *in vitro* cellular responses, leading to the genesis of new tissue. This innovative study highlights the potential of electrostatic flocking as a powerful method for scaffold fabrication in vascular tissue engineering. However, the limited number of empirical studies in this field calls for further research to validate and build upon these initial findings.

### B. Wearable sensors

**FIG. 5.**
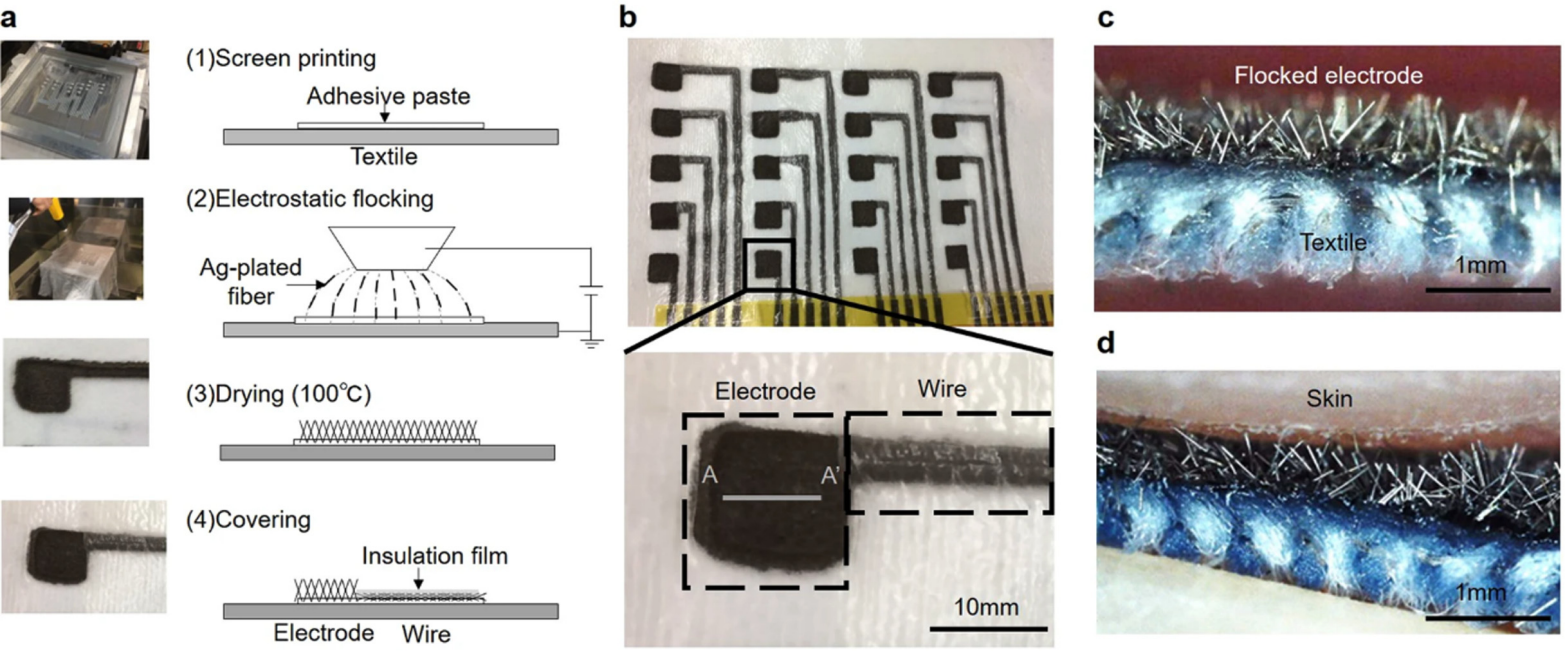
The image presents electrodes and wires created using electrostatic flocking technology. (a) The process of making electrodes and wires, where silver-plated fiber is flocked onto a textile surface using an adhesive paste. (b) A photograph of these electrodes and wires as they appear on the textile. (c) A cross-sectional view along the A-A′ line of the electrode. (d) The electrode upon contact with skin. Reproduced with permission from Takeshita *et al.*, Sci. Rep. **9**, 5897 (2019). Copyright 2019 Authors, licensed under a Creative Commons Attribution (CC BY) License.

The development of wearable sensors has led to significant advancements in healthcare technology. One notable innovation is the creation of a wearable multi-lead electrocardiogram (ECG) measurement apparatus as shown in Fig. 5 (Takeshita *et al.*, 2019). This device integrates electrodes and interconnecting circuitry directly onto a textile platform using silver-plated fibers. These fibers, with a diameter of 18 *μ*m, are meticulously aligned onto the textile substrate through the electrostatic flocking technique, ensuring consistent adherence and optimal functionality.

Unlike conventional systems that capture the standard 12-lead ECG signals during clinical assessments, the wearable apparatus demonstrated its proficiency in capturing an enhanced 18-lead ECG (Takeshita *et al.*, 2019). This advancement highlights the potential of wearable technology in providing more comprehensive cardiac monitoring outside clinical settings. This marks a significant leap in diagnostic capabilities. Furthermore, the resultant flocked wire exhibited commendable attributes in terms of elasticity and resilience against washing, which are practical imperatives for long-term use. A rigorous comparative analysis revealed a strong concordance between the ECG waveforms captured by this innovative system and those derived from established commercial systems.

The demonstrated efficacy of this work demonstrates the transformative potential of the electrostatic flocking technique in the domain of wearable sensors. However, it is imperative to acknowledge that although its efficacy underscores its potential, the nascent stage of this technology alludes to inherent limitations yet to be fully addressed. Section V C investigates the advantages and disadvantages in the application of electrostatic flocking.

### C. Importance and limitations of electrostatic flocking

Electrostatic flocking, a cutting-edge textile technology, has emerged as a promising approach in tissue engineering and regenerative medicine, potentially outpacing some traditional methodologies. Recent studies have highlighted the potential of flocked materials to offer favorable *in vitro* and *in vivo* responses, largely due to their customizable porosity and fiber density (McCarthy *et al.*, 2021). The intrinsic design of these materials, characterized by parallel fibers aligned perpendicular to the substrate, provides scaffolds with high porosity and robust mechanical integrity (Ribeiro *et al.*, 2017). This alignment creates fibrous assemblies with a high surface-to-volume ratio, which significantly enhances cellular adhesion, leading to beneficial effects for cell culture (Vellayappan *et al.*, 2015).

One of the key advantages of electrostatic flocking is the ability to control fiber density and scaffold porosity. This control is achieved by adjusting the length of the fibers and the duration for which voltage is maintained at the charging electrode, a process commonly referred to as “flock time” (McCarthy *et al.*, 2021). By fine-tuning these parameters, researchers can create scaffolds tailored to specific applications, optimizing their mechanical and biological properties.

However, it is crucial to recognize the challenges and limitations associated with electrostatic flocking. The main challenge lies in the stringent requirements for fiber electrical conductivity, which can complicate the production process. Additionally, producing discrete flock fibers can be difficult, and there is a relative lack of comprehensive understanding surrounding the methodology and synthesis of these materials (McCarthy *et al.*, 2021). Despite its potential, flocking is still in the early stages of development for tissue engineering applications, and further research is needed to fully realize its capabilities (Tonndorf *et al.*, 2021).

Comparing electrostatic flocking with traditional methods, the former offers significant advantages in terms of customization and functionality (Gossla *et al.*, 2021). However, it also faces notable hurdles that need to be addressed. The need for highly conductive fibers and the complexities of the production process are significant obstacles (McCarthy *et al.*, 2021). Moreover, the limited understanding of the underlying principles and synthesis techniques poses a challenge to the broader adoption of this technology.

## VI. WEAVING: A MULTIFACETED APPROACH IN MATERIAL ENGINEERING

**FIG. 6.**
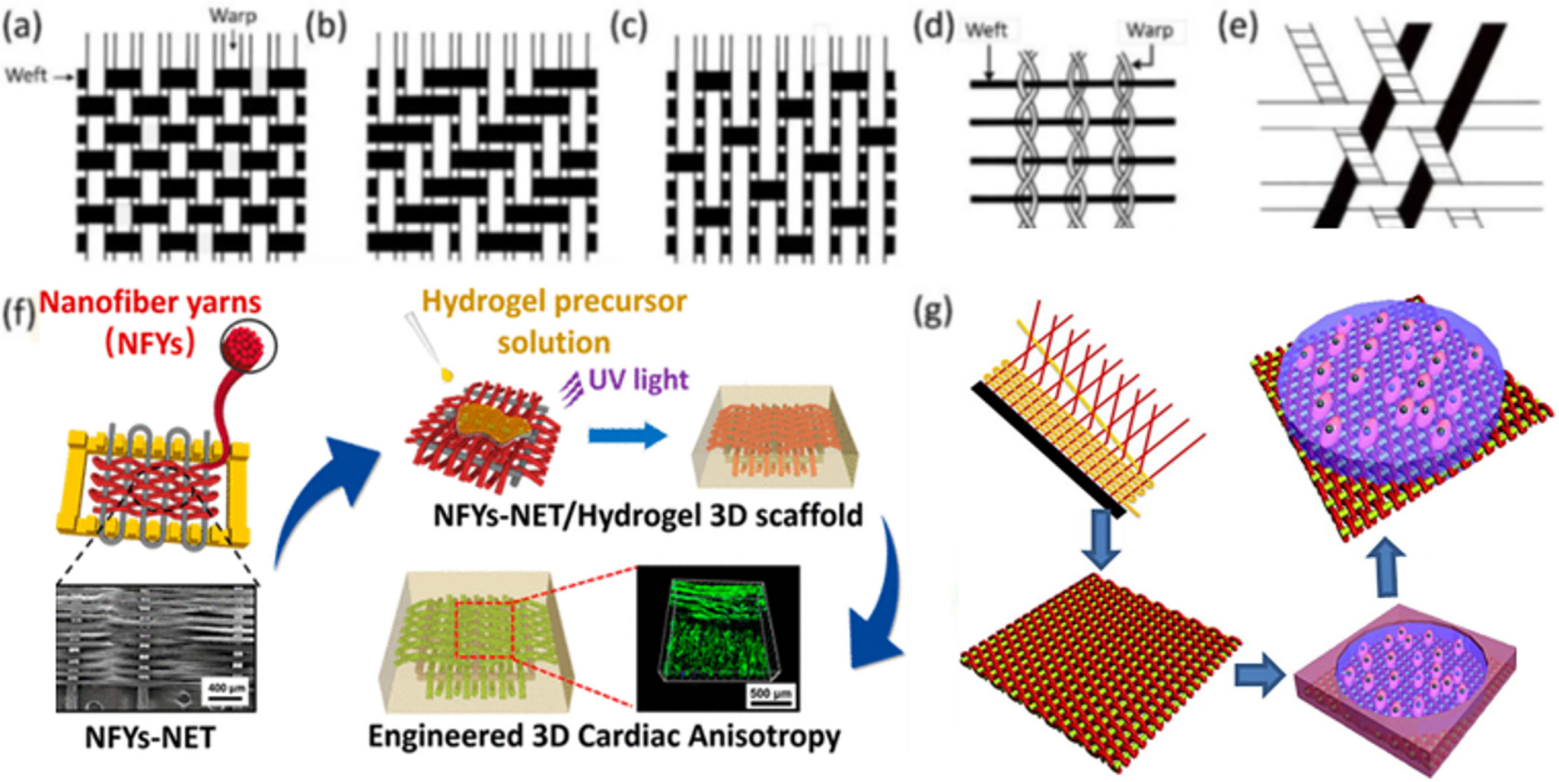
The image illustrates several woven patterns: (a) plain, (b) twill, (c) satin, (d) leno, and (e) triaxial weaving. Additionally, it shows (f) a composite scaffold created by combining hydrogel with woven fabrics for cardiac tissue engineering. Reproduced with permission from Wu *et al.*, ACS Nano **11**(6), 5646–5659 (2017). Copyright 2017 American Chemical Society. (g) A composite scaffold made by integrating cell-laden hydrogel with woven fabrics for heart valve engineering. Reproduced with permission from Wu *et al.*, Acta Biomater. **51**, 89–100 (2017). Copyright 2017 Elsevier.

Weaving involves the controlled interlacing of two sets of yarns on either manual or mechanized looms. This established method, deeply rooted in textile tradition, finds application in biomedical engineering, fuel cell technologies, and composite material manufacturing (Akbari *et al.*, 2016). A basic woven pattern is plain, where each weft passes over one warp and then under the next, alternating in each row [Fig. 6(a)] (Wu *et al.*, 2017). Despite its simplicity, the plain pattern allows for adjustments to fabric properties. By varying the weaving density of warps and wefts, researchers can control the fabric's strength, stability, and porosity, which in turn affect the degradation rate and cell distribution of scaffolds. Different materials can be used for warps and wefts to mimic the anisotropic properties of tissues such as bone and cardiac tissues. Heavyweight yarns can create a raised rib in the fabric, resembling striated tissues such as cardiac and skeletal muscles. Twill and satin patterns are modifications of the plain pattern. In twill, a weft passes over and under multiple warps in an alternating sequence, creating a diagonal pattern on the fabric surface [Fig. 6(b)]. In satin, a warp floats over four or more wefts before passing under one weft and repeating the process [Fig. 6(c)]. Twill and satin fabrics are less stable than plain fabrics but have higher tear strength due to the ease with which yarns can move and bunch together. Satin fabrics are asymmetrical, with warps predominantly on one face and wefts on the opposite side, useful when differing cell affinity or mechanical properties are needed on each side. Leno and triaxial woven patterns have gained importance in technical textiles. In leno, two warps twist and grip around the weft, providing more stability than plain fabrics [Fig. 6(d)]. Triaxial patterns interlace three yarn sets in three directions, optimizing shear resistance and making them suitable for constructing scaffolds for cardiac tissues [Fig. 6(e)]. Another similar pattern, the tetra-axial pattern, consists of four sets of yarns inclined at 45° to each other. Two-dimensional woven fabrics, particularly plain fabrics, have been used as scaffolds in tissue engineering. To improve biocompatibility and thickness, hydrogels can be integrated with woven fabrics to create composite scaffolds [Figs. 6(f) and 6(g)] (Wu *et al.*, 2017). However, these composite scaffolds tend to delaminate into layers and are unsuitable for tissues experiencing multidirectional stresses. Utilizing three-dimensional weaving for scaffold fabrication can address some of these limitations (Jiang *et al.*, 2021).

Making woven-based scaffolds remains predominantly a manual method (Babu *et al.*, 2021), which limits both the precision of manufacture and throughput. While the fundamental principles of weaving remain constant, manufacturing methods have evolved and become more sophisticated to adapt to varying requirements, whether for heightened efficiency, delicate material manipulation, or the inception of intricate patterns (Liberski *et al.*, 2017).

The characteristics of woven fabrics, such as tensile strength, porosity, thickness, flexibility, and resilience, are contingent on multiple factors, including the choice of weave pattern, thread density, and the inherent properties of the raw materials (Liberski *et al.*, 2017). Further, customizing fabric attributes can be achieved through manipulating the warp-weft density, yarn gauge, and interlacing parameters.

While many woven patterns have been devised over the past seven millennia, plain, twill, and satin weaves dominate as the principal 2D scaffold structures (Fig. 6). In plain weave, alternate interlacing creates a robust but less flexible fabric. Conversely, the diagonal pattern of the twill weave enhances elasticity, while the satin weave, with its minimal interlacing points, ensures a softer texture, albeit at the cost of wear resistance (Jiao *et al.*, 2020).

**FIG. 7.**
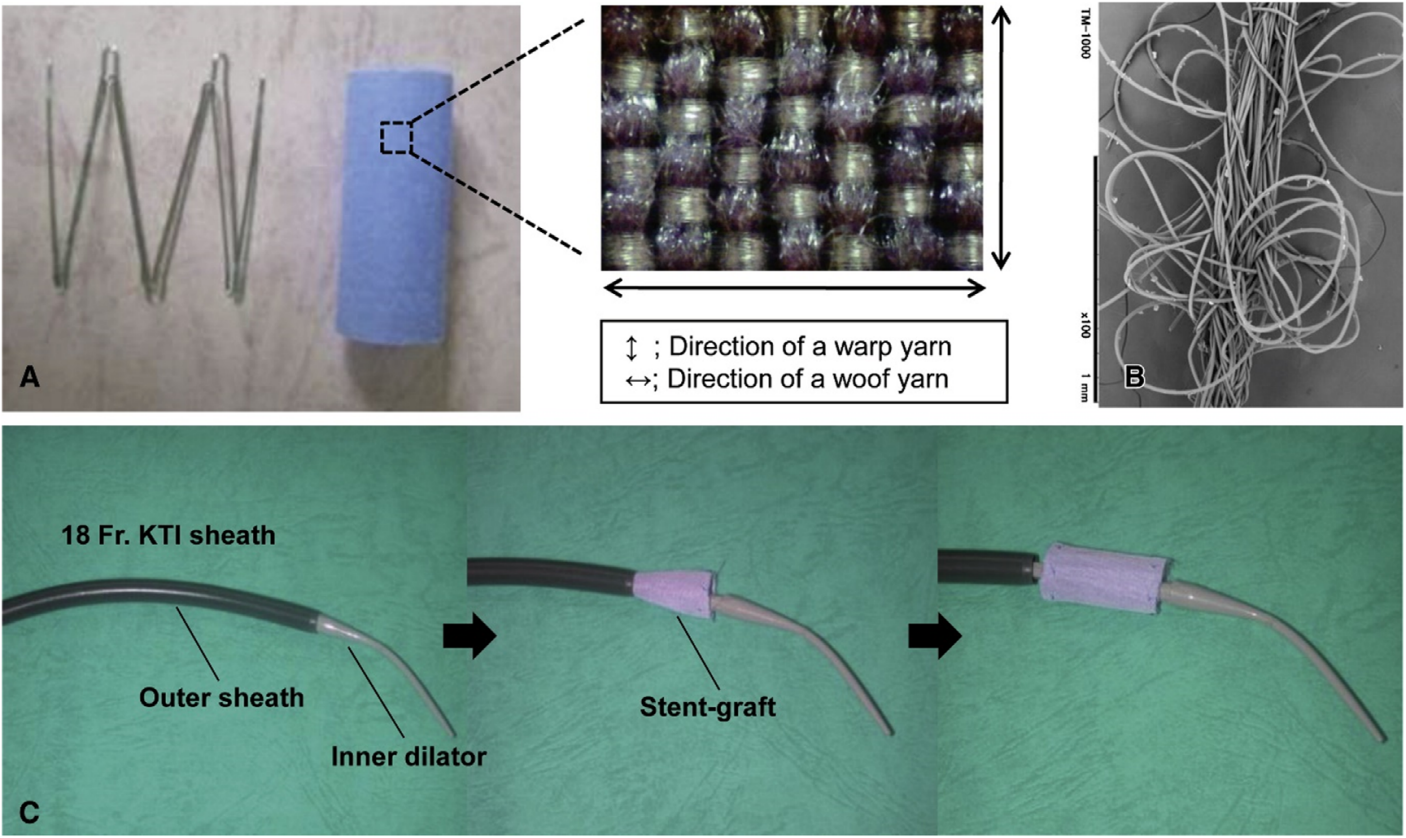
(a)–(c) A stent-graft showcasing the application of weaving in biomedical engineering. (a) The stent-graft, woven with polyethylene terephthalate (PET) and polyglycolic acid (PGA), highlighting the integration of traditional weaving with advanced materials. (b) The stent-graft's double-layered warp yarn, emphasizing the precision and complexity in medical textile design. (c) The delivery system, the ^18^F Keller-Timmermann Introducer Sheath (KTI), illustrating the combination of woven structures in sophisticated medical devices. This figure exemplifies the innovative use of weaving techniques in developing essential biomedical tools. Reproduced with permission from Takeuchi *et al.*, J. Thoracic Cardiovasc. Surg. **148**, 1719–1725 (2014). Copyright 2014 Authors, licensed under a Creative Commons Attribution (CC BY) License.

Adding another dimension to this fabric tapestry is the tubular 3D weave. Characterized by void spaces, these woven designs can closely emulate the fibrous architecture of various native tissues, including myocardial tissue (Akbari *et al.*, 2016). Bifurcated, circular tubular weaves have been suggested to replicate the vascular networks intrinsic to human physiology (Liu *et al.*, 2018). Such designs have found application as vascular scaffolds (Singh *et al.*, 2015). Furthermore, honeycomb-structured tubular weaves, which can closely mirror the mechanical attributes of the human myocardium, hold promise for cardiac tissue engineering (Liao *et al.*, 2013). Augmenting these woven constructs with hydrogels or cell-hydrogel amalgamations can further accentuate their biocompatibility. Potential applications extend to cardiac patches, vascular grafts, stents, and even pacemakers. Figure 7 demonstrates a stent-graft using weaving in biomedical engineering (Takeuchi *et al.*, 2014). This figure exemplifies the innovative application of weaving in medical devices. In-depth explorations into these weaving applications will be presented in Secs. VI A–VI C.

### A. Cardiac patch

Recent studies describe the innovative approach of employing woven nanofiber yarns to fabricate cardiac patches. Significant research has been conducted, with findings consistently affirming the utility of these woven scaffolds in biomedical applications (Babu *et al.*, 2021; Sunil Richard and Verma, 2022).

A recent study introduced a 3D-woven scaffold made from polymer nanofibrous yarn, which was encapsulated with α-mangostin, a phytochemical known for its cardioprotective, anti-inflammatory, and antioxidant properties. The scaffold was created through a controlled process where electrospun nanofibers were twisted into yarns and woven to mimic textile structures. By using a 12% PCL solution and adjusting the collector rotation speeds to 400, 500, and 600 rpm, different nanofiber yarns were produced. The yarn spun at 600 rpm was found to have the best stability for further processes. This woven fabric patch, sized 10 × 5 cm^2^ (50 cm^2^), was then treated in a methanolic α-mangostin bath. *In vivo* tests showed that the scaffold could enhance angiogenesis, improve tissue compatibility, and support the growth and function of cardiac stem cells, suggesting its potential in cardiac tissue engineering (Sunil Richard and Verma, 2022).

Another innovative approach involved the design and development of interwoven electrospun nanotextile patches using poly-l-lactic acid (PLLA) and poly-caprolactone/collagen (PCL/Col). The process began with the electrospinning of nanofibrous yarns, which were then subjected to heat-stretching and plying to enhance their mechanical properties. These yarns were interwoven using a plain weaving technique, resulting in tightly packed patches. The resulting nanotextile demonstrated strong mechanical properties, anisotropic behavior, and excellent hydrophilicity. Additionally, when endothelial cells were seeded onto the woven patches, they exhibited superior cell adhesion and proliferation. The nanotextile patches also maintained their structural integrity during *in vitro* tests, showing promise for use in vascular tissue engineering applications (Babu *et al.*, 2021).

### B. Vascular grafts

Vascular grafts using woven nanofiber yarns are being developed. Several comprehensive studies across different types of research have been conducted and will be elaborated on in the following paragraphs.

A groundbreaking silk-based bilayered vascular conduit of small diameter has been developed, featuring enhanced mechanical strength and cellular properties (Krishnan *et al.*, 2023). Utilizing a combination of cylindrical weaving and electrospinning techniques, a seamless bilayered conduit with a 3 mm diameter was synthesized. This conduit comprises an inner nanofibrous layer made of poly-caprolactone/collagen, which supports cellular compatibility, and an outer woven silk layer that provides mechanical compliance. The electrospinning process employed a 20% (w/v) poly-caprolactone–collagen blend (3:1) in TFE (1,1,2-trifluoroethanol) to create the inner layer. Compared to industry standards, this bilayered conduit demonstrated superior mechanical performance, including increased burst strength, suture retention, compliance, and leak resistance. Initial *in vitro* tests suggest that the conduit lumen is non-hemolytic and promotes endothelial cell adhesion and viability. These findings highlight the potential of this bilayer scaffold for use in small diameter vascular prosthetics.

Additionally, a novel approach to fabricating woven nanotextiles from low-strength nanoyarns for vascular applications has been developed (Joseph *et al.*, 2018). The process involved electrospinning 14% poly-l-lactic acid (Mw = 100–140 kDa) dissolved in a chloroform and acetone mix (3:1 ratio), and 20% w/v poly-caprolactone (Mw = 45 kDa) blended with collagen in 1,1,1,2-tetrafluoroethane (3:1). The yarns were assembled using a plying technique in preparation for weaving. This unique woven nanotextile structure induced significant hydrophilic behavior in the inherently hydrophobic material, which enhanced protein adsorption and subsequently promoted cell adhesion and proliferation. To validate the efficacy of the nanotextile conduit as a potential arterial embolizer, graft implantation in rabbits was conducted, examining attributes such as strength, suture retention, kink resistance, and non-thrombogenic properties.

A study explored the practical significance of integral water permeability in textile vascular grafts. The research aimed to address the correlation between water permeability and blood permeability, which has not been clearly established (Guan *et al.*, 2021). Four types of woven vascular grafts were designed and tested for their permeability to water, simulated plasma, and anticoagulated whole blood at varying pressures. The study found steady correlations between water and blood permeability, suggesting that evaluating blood permeability using water permeability measurements is feasible. This method provides a quantitative approach for guiding the design and pre-clotting operations of textile vascular grafts. These findings highlight the potential of using water permeability as an objective measure to predict blood permeability, thus offering practical significance for the development and clinical application of textile vascular grafts.

### C. Vascular stents

A seamless tubular woven bifurcate stent graft has been developed, utilizing silk fibroin to enhance graft patency through an advanced medication modification procedure (Liu *et al.*, 2019). This process involves weaving grafts from degummed silk, raw silk, and polyethylene terephthalate in a carefully controlled environment. Experiments with human vascular smooth muscle (HVSM) cells near these modified grafts showed a significant reduction in HVSM cell proliferation. With attributes such as appropriate graft thickness, resistance to water permeability, and anti-occlusion functionality, the heparin-functionalized bifurcated stent graft is poised to be an effective tool for vascular disorders.

A robust woven vascular stent-graft has been engineered with excellent mechanical properties, biocompatibility, and enhanced drug delivery capabilities aimed at mitigating thrombosis and controlling the expansion of abdominal aortic aneurysms (Liang *et al.*, 2023). Using an emulsification-precipitation method, paclitaxel (PTX) and metformin (MET)-loaded silk fibroin microspheres were created, self-assembling and adhering electrostatically in layers on the stent surface. The production began with precipitating a solution containing silk fibroin, polyethylene glycol, and methanol in specific proportions. Silk fibroin microspheres were generated through cycles of centrifugation, ultrasonic dispersion, and lyophilization. For drug-infused microspheres, metformin and paclitaxel were dissolved in respective solutions and combined in a precise ratio to optimize drug delivery. The results showed that these microspheres improved drug release efficacy due to their enlarged specific surface area, with a prolonged release trajectory of over 70 h and minimal water permeability. The combination of PTX and MET effectively inhibited the proliferation of human umbilical vein endothelial cells. This study highlights the potential of dual-drug-loaded woven vascular stent-grafts to significantly enhance therapeutic interventions for abdominal aortic aneurysms.

### D. Importance and limitation of weaving

Woven textiles are renowned for their superior strength and heightened stability relative to other fabric formations with interlaced fibers (El-Ghazali *et al.*, 2022). Intrinsically, the weaving process yields structures with distinct anisotropic properties. Although weaving imposes lesser mechanical strains compared to knitting, the resultant fabrics exhibit reduced porosity and flexibility (Wu *et al.*, 2020). Additionally, the pore size in woven fabrics is relatively smaller than that in knitted counterparts, which can be advantageous or limiting depending on the intended application.

For biomechanical applications necessitating robust load-bearing capabilities, the interlocking of multiple woven layers can fortify in-plane mechanical properties (Lu *et al.*, 2024). This layered approach facilitates the fabrication of dense three-dimensional (3D) structures, enabling the emulation of the mechanical attributes inherent to certain biological tissues, such as cardiac tissue and cartilage (Liberski *et al.*, 2017).

Notably, the construction of weaving looms is less intricate than knitting systems, offering an avenue for the streamlined production of cell-embedded structures with predefined mechanical characteristics and cell distributions (Pedde *et al.*, 2017). The versatility of textile-based techniques, encompassing both weaving and knitting, is underscored by their use in the fabrication of diverse medical devices, including PGA nerve grafts, PET vascular grafts, and warp-knitted PET meshes for cardiac support (Senel-Ayaz *et al.*, 2018).

Knitted fabrics, distinguished by their well-defined loop structures, offer notable elasticity (Haroglu, 2022). The versatile potential of these fabrics allows for tailored porosity and mechanical properties by modulating yarn parameters and knitting configurations. Section VII delves deeper into the specific applications of knitting within cardiovascular contexts.

## VII. KNITTING

**FIG. 8.**
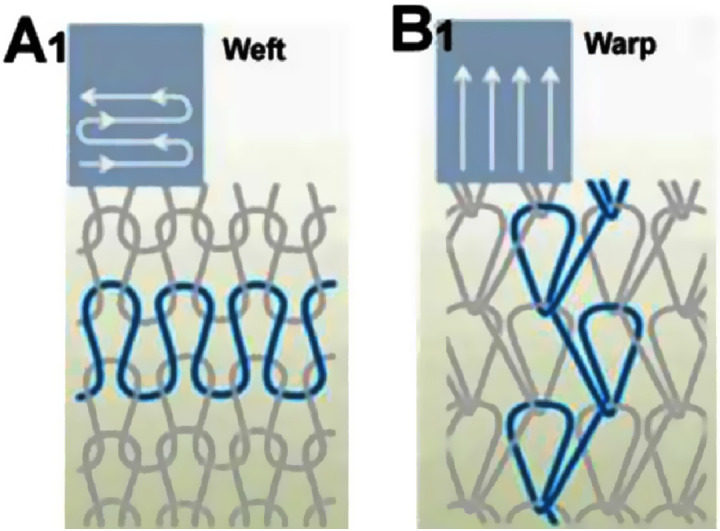
Comparison of weft and warp knitting structures: (A1) weft structure and (B1) warp structure. Reproduced with permission from Liberski *et al.*, Global Cardiol. Sci. Pract. **2016,** e201631 (2016). Copyright 2016 Authors, licensed under a Creative Commons Attribution (CC BY) License.

**FIG. 9.**
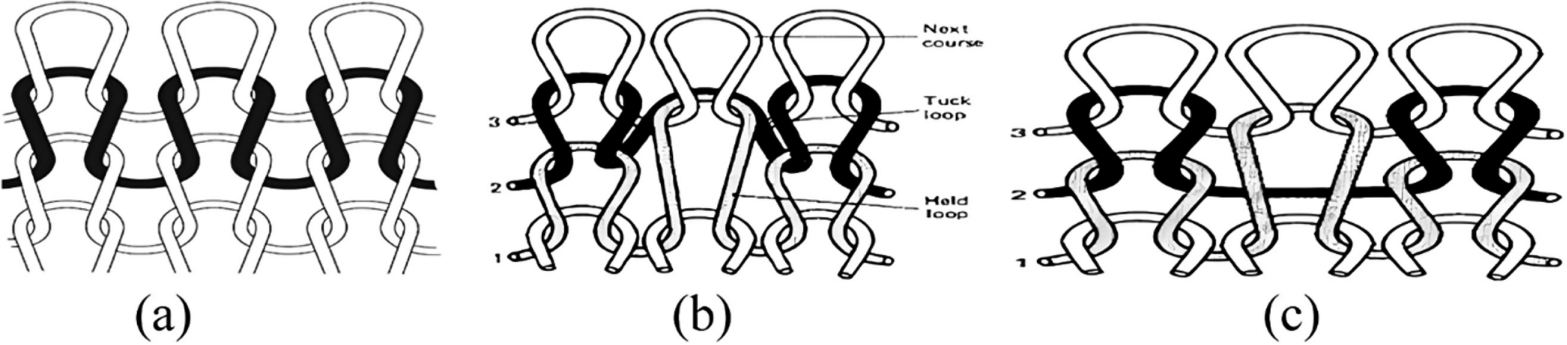
Basic types of stitches in knitting process (a) knit stitch, (b) tuck stitch, and (c) float stitch. Reproduced with permission from Assefa and Govindan, J. Eng. Fibers Fabrics **15**, 1558925020928532 (2020). Copyright 2020 Authors, licensed under a Creative Commons Attribution (CC BY) License.

Knitting is a traditional textile technique capable of creating intricate 2D and 3D designs by interlocking yarns into carefully structured loops. The process involves threading yarns through previous loops, forming a continuous series of interconnected loops (Mishra, 2023). There are two main types of knitting: weft and warp (Girault *et al.*, 2021). In weft knitting, stitches from a single yarn align horizontally, creating cohesive rows or courses, each building upon the previous one. In warp knitting, multiple parallel yarns are looped vertically (Fig. 8) (Liberski *et al.*, 2016). In knitting terminology, a “wale” refers to a vertical column of stitches, while a “course” denotes a horizontal row. Warp knits typically not only have a flat, smooth surface but can also be textured with a pile. They offer varying crosswise elasticity and minimal vertical extensibility and are produced in various weights and fiber types. Warp knits resist unraveling and run due to their interlocked threads. Weft knits, on the other hand, exhibit significant longitudinal stretch and moderate to high crosswise elasticity (Khademolqorani *et al.*, 2021). Figure 9 shows common knit stitches, which include the plain, purl (or reverse-knit), missed/float (resulting in yarn floats on the fabric's reverse), and tuck stitches, which create openings in the fabric (Assefa and Govindan, 2020). Warp textiles generally possess greater tensile strength than weft fabrics due to their robust interlocked threads.

Knitted structures are characterized by their course and wale, determined by the number of rows spanning their width and length, respectively. Wale density within a fabric length is influenced by factors such as needle diameter (known as “gauge”), yarn properties, and the tension applied to the yarn during fabrication (Kabir *et al.*, 2023). The resulting structures exhibit a complex interplay of mechanical and physicochemical properties, depending on the knitting method, stitch architecture, and yarn type. Tuck stitches, for instance, produce fabrics that are more expensive, denser, and slightly less elastic than standard stitches, while also increasing porosity (Akbari *et al.*, 2016). Due to their inherent elasticity and adaptability, knitted textiles have become significant in tissue engineering applications (Lin *et al.*, 2020). However, their large pore sizes can be suboptimal for cell seeding. Recent research has combined materials such as collagen (Pu *et al.*, 2016), chitosan (Dumont *et al.*, 2016), silk sponges (Fan *et al.*, 2009), and electrospun fibers (Han *et al.*, 2021) to create composite scaffolds with excellent mechanical and biological properties. In therapeutic settings, knitted scaffolds are widely used to repair or regenerate damaged tissues and organs (Liberski *et al.*, 2016). Compared to other biotextile manufacturing methods, knitting incorporates a more intricate yarn structure, offering designs with greater complexity and functional versatility.

### A. Cardiac patches

Recent advancements in biotextile engineering have significantly advanced the fabrication of knitted cardiac patches. Such innovations in cardiac tissue engineering have shown promising results, proving the viability of knitted scaffolds for tissue regeneration.

One notable study demonstrated the biological efficacy of three-dimensional (3D) knitted scaffolds for cardiac tissue engineering by creating 3D scaffolds using polyethylene terephthalate (PET) in a pile loop knit configuration (Haroglu *et al.*, 2022). These scaffolds were inoculated with 2 × 10^5^ C_2_C1_2_ murine myoblast cells for *in vitro* experiments. The cell culture medium was enriched with 4500 mg/l glucose, 10% fetal bovine serum (FBS), 1% l-glutamine, and a 1% mix of penicillin-streptomycin. The biomechanical properties of these scaffolds, as measured by Young's modulus, were comparable to human myocardial tissue. The anisotropic porous structure of the knitted scaffold enhanced the attachment and proliferation of the C_2_C1_2_ murine myoblast cells. The high surface-to-volume ratio of the scaffolds, along with optimal porosity, significantly promoted cellular adhesion and proliferation, indicating substantial potential for regenerative medicine and tissue engineering.

Another study explored two distinct knitted polytetrafluoroethylene (PTFE) scaffolds (Terakawa *et al.*, 2022). The first scaffold was created from finely knitted PTFE fibers compressed to increase the density of the PTFE particulates. The second scaffold, termed K-PTFE, was made from thin PTFE threads interwoven with micro-PTFE particulates. This study included a control group using commercially available expanded PTFE (ePTFE) patches. Male micromini pigs, weighing between 25 and 30 kg, were used as biological models for these scaffold implants. The evaluation revealed that tensile properties, intimal hyperplasia thickness, and endothelialization rates were comparable across the three patch types. However, the novel PTFE patches showed reduced macrophage infiltration compared to the control ePTFE and also lower infiltration of macrophages, lymphocytes, and granulocytes outside the patch. Over extended periods, there was a notable decrease in cellular penetration outside these new patches, with both novel patches showing similar results. These findings suggest that the new knitted PTFE patches not only match the durability and resilience of commercially available ePTFE patches but also reduce inflammatory responses.

### B. Vascular grafts

**FIG. 10.**
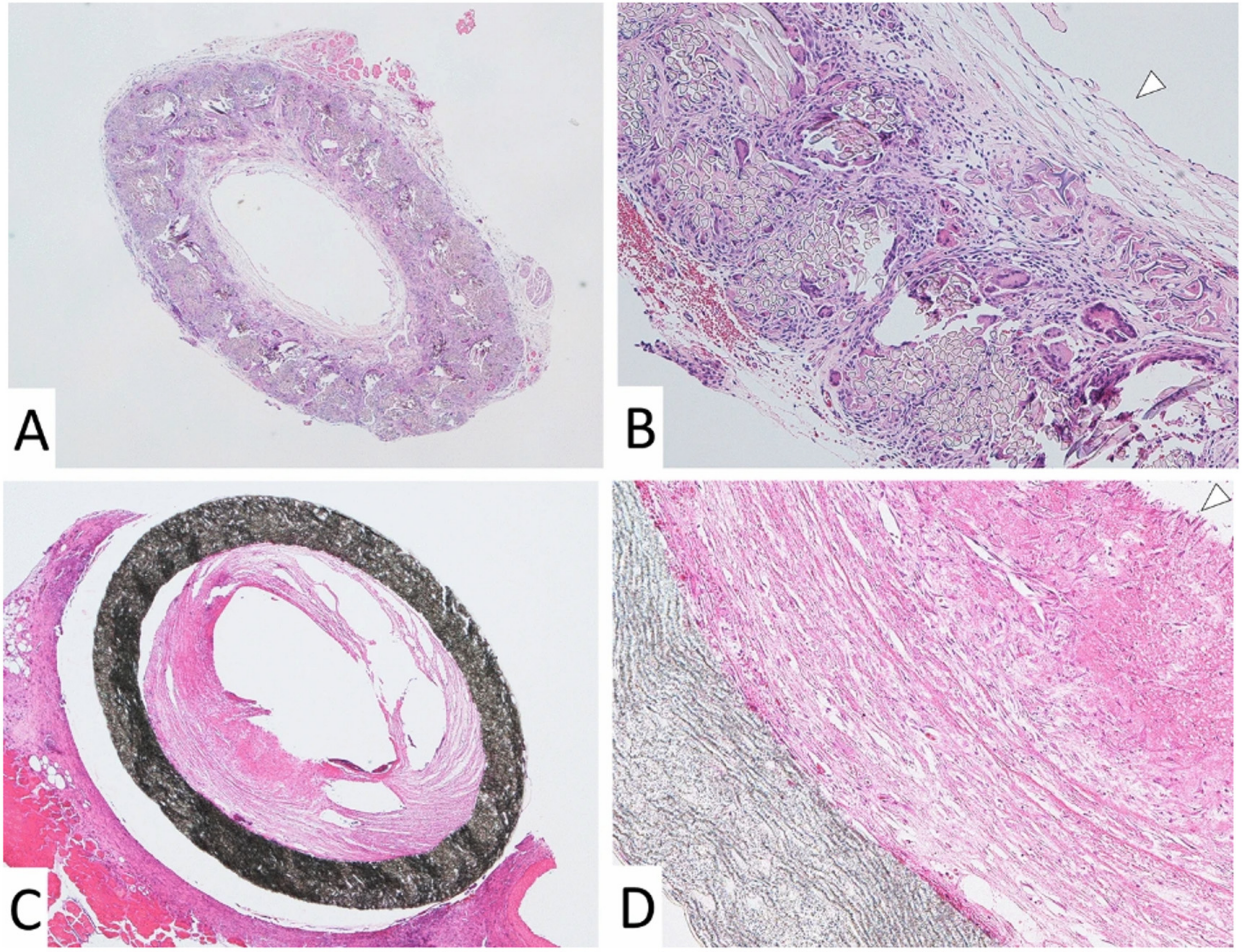
The image illustrates microscopic comparisons of vascular grafts. (a) and (b) A double-Raschel knitted silk fibroin (SF) graft at 40× and 100× magnification, respectively, showing a fully patent lumen and cellular integration. (c) and (d) An expanded polytetrafluoroethylene (ePTFE) graft at similar magnifications, revealing a narrowed lumen and fibrin accumulation without cellular coverage. This contrast underscores the distinct microstructural properties of knitted SF and ePTFE materials in vascular grafts. Reproduced with permission from Kiritani *et al.*, Sci. Rep. **10**, 21041 (2020). Copyright 2020 Authors, licensed under a Creative Commons Attribution (CC BY) License.

The development of advanced knitting technologies for creating vascular grafts intended for tissue regeneration marks a significant advancement in the biomedical field (Wang *et al.*, 2022). Extensive research on knitted vascular grafts has shown their potential for cardiovascular interventions and tissue engineering. Comparative studies have highlighted the microstructural differences between knitted silk fibroin (SF) and expanded polytetrafluoroethylene (ePTFE) vascular grafts (Fig. 10), with microscopy revealing the knitted SF graft's fully patent lumen and cellular integration, in contrast to the narrowed lumen and lack of cellular coverage in ePTFE grafts (Kiritani *et al.*, 2020). The biomechanical properties and biocompatibility of knitted constructs made from polylactic acid (PLA) filaments have been quantified, focusing on their suitability for small-diameter vascular applications (Lou *et al.*, 2015).

Further analysis examined the impact of different wrapped yarns derived from poly(ethylene terephthalate) (PET) and polyether-polyurea copolymer (supplied as Spandex) on the biomechanical properties of vascular grafts (Lou *et al.*, 2016). The yarns were woven into various textile configurations, including braids, warp knits, and weft knits. An 11% polyvinyl alcohol (PVA) solution was integrated through a freeze–thaw method, transforming the knitted constructs into vascular grafts, which were then tested for circumferential properties and bursting tenacity. The study used PET multifilament yarns (75D/144F), Spandex filaments (70D), and high molecular weight PVA, with tissue from porcine carotid arteries as the biological model for comparison. The results indicated a direct correlation between graft compliance and bursting strength with the frequency of freeze–thaw cycles. The inclusion of Spandex fibers enhanced the compliance of the vascular grafts and the overall textile structure.

This body of work highlights the potential of knitted vascular grafts to match the biomechanical properties of native vessels, underscoring their viability in cardiovascular and tissue engineering applications.

### C. Vascular stents

Knitting technology plays a pivotal role in the development of vascular stents, offering a versatile and effective method for creating structures that meet the rigorous demands of cardiovascular applications. Researchers explored the architectural composition and biomechanical properties of an innovatively fabricated reticulate, tubular polyvinyl alcohol (PVA) vascular stent (Ueng *et al.*, 2016). The fabrication process involved dual-layered knitting of PVA fibers, followed by immersion in an 80-kDa poly-caprolactone solution and thermal treatment. The resulting materials underwent *in vitro* degradation in a phosphate buffered saline milieu (PBS, pH = 7.4). The empirical results indicated that combining a knit loop architecture with a poly-caprolactone (PCL) film attached to the fibers produced elastic and compression resistant PVA vascular stents. The *in vitro* degradation trajectory confirmed the preservation of the structural path, validating the successful creation of the intended elastic PVA vascular stents.

Building on this work, another team investigated the morphological, mechanical, and biological attributes of composite stents, focusing on the effects of varying concentrations of poly-caprolactone (PCL) and polyethylene glycol (PEG) (Lin *et al.*, 2020). Their fabrication method involved mixing twisted poly-caprolactone (80 kDa) and polyethylene glycol (4 kDa) with biodegradable polyvinyl alcohol (PVA, 75D/25f) yarns before coating and thermal treatment. These coated yarns were then woven into composite stents with a core–shell morphology. Experimental data demonstrated that the flexibility of PVA yarns and the elasticity of weft knits were successfully integrated into the composite stents. The addition of PEG significantly enhanced the operational efficacy of these composite stents. Given the biodegradable nature of the materials and the structural integrity of the resultant stents, they show promise for use in cardiovascular interventions.

### D. Wearable sensors

Knitting technology has become a crucial method for developing wearable sensors, providing unparalleled flexibility, comfort, and seamless integration of electronics into textiles. The process begins with the selection of conductive yarns, such as silver-coated fibers, which are intricately knitted into fabrics to form the base of the wearable sensors (Stoppa and Chiolerio, 2014). These conductive textiles are essential for creating garments capable of monitoring various physiological parameters. The elasticity and breathability of knitted fabrics make them particularly suitable for continuous health monitoring applications (Tyler *et al.*, 2019). They allow sensors to conform closely to the body, ensuring accurate data collection while maintaining user comfort. The integration of conductive fibers into knitted structures can assist with the make of embedding electronic components necessary for data processing and transmission (Tseghai *et al.*, 2020).

One of the primary advantages of using knitted structures in wearable sensors is their inherent ability to incorporate miniaturized electronic components seamlessly (de Mulatier *et al.*, 2018). These components are integrated directly into the fabric, resulting in a sensor system that is both functional and discreet. Advancements in conductive yarns and fabrication technologies have opened new possibilities for designing and knitting seamless garments equipped with sensors. For example, this technique has been used to produce wearable antennas that function as strain sensors by leveraging the intensity variations of backscattered power from an inductively coupled RFID tag under physical stretching (Patron *et al.*, 2016). Additionally, computerized flatbed knitting methods have been employed to create elliptical waveguides, which are conductive textile sleeves filled with knitted polyester and use a silver-coated polyamide conducting yarn (Jia *et al.*, 2014).

In another research, an ultra-stretchable, comfortable, and single-layer triboelectric all-yarn-based all-textile sensor (AATS) with an innovative alternating high elastic knitting structure has also been developed (Si *et al.*, 2022). This AATS exhibits excellent electrical performance while maintaining the basic characteristics of commercial textiles. It combines mature seamless knitting techniques to integrate seamlessly into knitwear, offering promising aesthetics and comfort. Such smart garments, equipped with AATS, can provide unconstrained continuous breathing signal acquisition and sleeping position determination. Research has also demonstrated various innovative approaches to improve the performance of thread-type sensors, such as wrapping composite core yarns with piezoresistive fibers and utilizing double-twisted smart fibers (Meena *et al.*, 2023). Despite the remaining challenges in enhancing electrical performance and sensitivity, the integration of knitting technology with advanced materials and designs continues to expand the possibilities for wearable sensors.

These examples highlight the versatility and potential of knitting technology in creating advanced wearable sensors that are both functional and comfortable for everyday use. The ability to incorporate electronic components directly into the textile structure during the knitting process makes this approach particularly valuable for the rapid prototyping and production of smart clothing and wearable textiles.

### E. Importance and limitations of knitting

Knitting techniques in the realm of biomaterial applications, particularly for cardiovascular uses, offer significant benefits due to their unique structural properties. Knitted structures are known for their breathability, flexibility, and comfort, which are crucial for biomedical applications such as heart patches and vascular grafts (Jiao *et al.*, 2020). The porous nature of knitted fabrics enhances drug delivery by allowing efficient transport of therapeutic agents. Furthermore, the ease of customization with advanced knitting technologies enables the production of tailored biomedical devices that can meet specific patient needs, thereby improving the effectiveness of treatments. These advantages underscore the growing importance of knitted biomaterials in cardiovascular applications.

However, there are notable limitations to the use of knitted fabrics in this field. One of the primary challenges is ensuring the strength and durability of knitted structures, which may not always match that of woven or non-woven counterparts. The potential for piling and stretching of knitted materials can also be a concern, particularly in applications where high mechanical stability is required (Jiang *et al.*, 2021). Additionally, the production of complex shapes and sizes using knitted fabrics can be technically demanding and may require sophisticated machinery and expertise. Despite these limitations, ongoing advancements in knitting technology and material science continue to mitigate these challenges, paving the way for broader and more effective applications of knitted biomaterials in cardiovascular medicine.

## VIII. BRAIDING

**FIG. 11.**
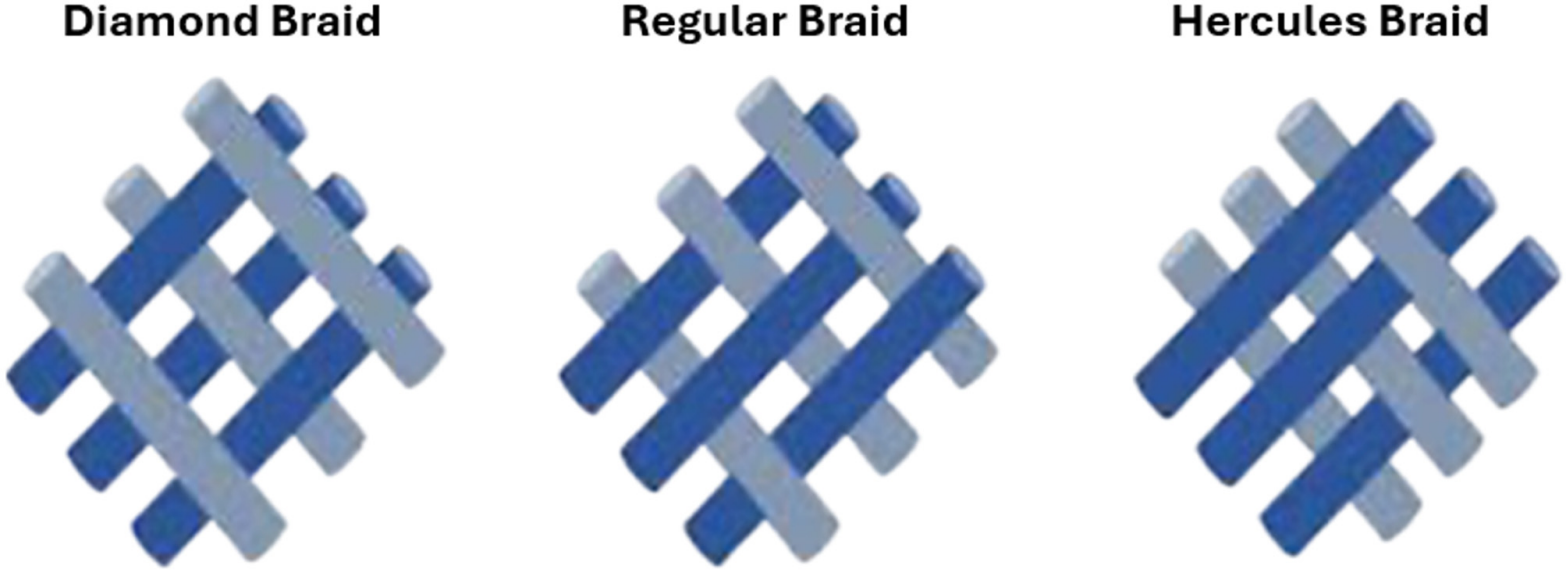
Three typical tubular 2D braids: Diamond braid features the highest interlacing density with a 1/1 intersection repeat (yarn continuously alternating over and under one yarn). Regular braid follows a 2/2 pattern, and Hercules braid a 3/3 pattern. Reproduced with permission from Doersam *et al.*, Adv. Mater. Technol. **7**, 2101720 (2022). Copyright 2022 Authors, licensed under a Creative Commons Attribution (CC BY) License.

Employed for the creation of ropes and aesthetic hairstyling for five millennia, braiding today is a technique for forming intricate textiles by intertwining three or more yarns to engineer cylindrical or planar structures (Jiang *et al.*, 2021). Braiding has gained increasing attention in the development of biomedical applications for ligament, tendon, or cartilage tissues because of its high tensile strength (Tamayol, 2013). The three types of common braids (Diamond, Regular, and Hercules) are shown in Fig. 11 (Doersam *et al.*, 2022). Common medical applications of braided textiles are non-compliant metal stents, prostheses, sutures, and bandages. However, stiff metallic stents, in contrast to scaffold-engineered tissues, do not match the mechanical properties of the native tissue but brace the tissue open (Singh *et al.*, 2015).

Through controlled manipulation of obliquely interlaced strands, a diverse three-dimensional geometric construct with precise, stable attributes can be created. Medical textiles, such as sutures, vascular stents, and conduits tailored for nerve regeneration, can be produced using harness braiding technology (Chang *et al.*, 2020). The morphology of scaffolds, the fibers used, and the interactions between fibers play crucial roles in the optimal functioning of artificial grafts. Variation in braiding angles, fiber density, and layering allows the construction of scaffolds with anisotropic mechanical and physicochemical properties, offering an adjustable gradient in any chosen direction (Zbinden *et al.*, 2020). Optimizing porosity gradients, transitioning from the extremities toward the central region, bolsters cellular ingrowth while preserving mechanical integrity.

**FIG. 12.**
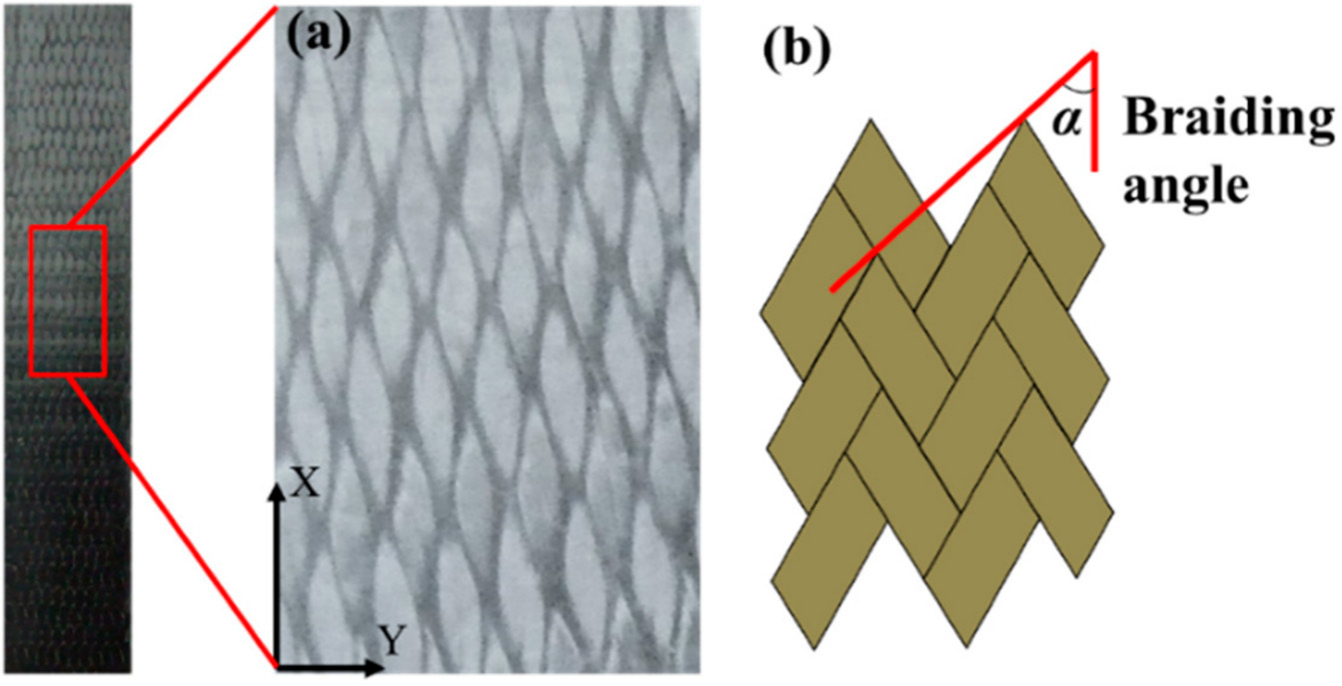
(a) Magnified view of a braided structure, illustrating the intricate interlacing of fibers along the X- and Y-axes. (b) Diagram showing the braiding angle (α), a critical parameter that influences the mechanical properties and stability of the braid. The precise configuration and alignment of fibers determine the overall performance and application suitability of the braided material. Reproduced with permission from Zhang *et al.*, Polymers **14**, 1916 (2022). Copyright 2022 Authors, licensed under a Creative Commons Attribution (CC BY) License.

Selecting appropriate fiber materials is essential in designing braided tissue constructs (John *et al.*, 2015). For instance, a composite biodegradable braided stent was created using poly (p-dioxanone) (PPDO) monofilament and PPDO/PCL multifilament to enhance mechanical strength and resistance to deformation (vessel recoil) (Sun *et al.*, 2019). This stent, made with 28 monofilaments and 4 multifilaments at a braiding angle (critical parameter that influences the mechanical properties and stability of the braid and shown in Fig. 12) of about 55°, was designed to improve yarn friction, prevent interlacing point slippage, and achieve the desired porosity for cell adhesion. The stent, with an internal diameter of 8 mm, was suitable for use in children's pulmonary arteries. *In vivo* evaluations confirmed that the braided structure had adequate properties and acceptable biocompatibility.

Another example includes the engineering of a tri-layer scaffold with an inner and middle layer made from electrospun silk fibroin (SF) and poly(l-lactide-co-ε-caprolactone) (PLCL), and an outer layer of braided SF yarn, to replicate the structure of native blood vessels (Zhang *et al.*, 2017). This design significantly increased the suture retention strength, bursting strength, compliance, and mechanical stability. Additionally, the prototype exhibited excellent mechanical properties, biocompatibility, and appropriate anticoagulation properties due to the heparin coating.

While biogenic materials create conducive ecosystems for cellular proliferation and activity, synthetic materials confer structural resilience (Mani *et al.*, 2024). Integrating nanofeatures into these structures amplifies and regulates cellular dynamics.

### A. Vascular graft

Braiding technology has emerged as a significant method in the development of vascular grafts, offering precise control over the mechanical and structural properties of the grafts. The intricate weaving of fibers allows for the creation of scaffolds that closely mimic the natural architecture of blood vessels, providing improved performance and compatibility for vascular applications.

One study demonstrated the creation of an intricate tri-layered vascular graft using sulfated silk fibroin, which involved braiding, freeze-drying, and silk solution coating processes (Ding *et al.*, 2016). The silk yarn was intertwined using a high-velocity rope braiding apparatus. Following the braiding, the tubes were enveloped in a matrix of sulfated silk fibroin combined with silk fibroin aqueous solution. The assembly was cryogenically stabilized at −20 °C for 12 h and then lyophilized for approximately 72 h. These trilayered constructs exhibited water permeability, tensile strength, burst pressure, and suture retention capabilities comparable to those of saphenous veins typically used in vascular grafts. *In vitro* evaluations confirmed their cytocompatibility. Recognizing their physical and biological efficacy, the study highlighted the potential of trilayered sulfated silk fibroin grafts for crafting small-diameter vascular grafts.

Another study examined the controlled variation of braiding parameters on vascular grafts (Zbinden *et al.*, 2020). The scaffolds were constructed from braided poly (glycolic acid) (PGA) fibers, enveloped with a poly (glycerol sebacate) (PGS) sheath. To critically assess the performance under different braiding configurations, these grafts were integrated into the infrarenal abdominal aorta of a Beige mouse model and monitored using 4D ultrasound diagnostics. After a 12-week implantation period, the vessels were examined for biaxial mechanical attributes and general histological constitution. Critical scaffold parameters such as braiding angle, fiber density, and the strategic inclusion of a PGS layer significantly influenced graft outcomes. Varying these factors altered inflammation gradients, extracellular matrix architecture, graft dilation, neovessel elasticity, and graft longevity. The researchers concluded that sculpted scaffolds with diverse property spectra can be optimized as tissue-engineered vascular grafts (TEVGs).

### B. Vascular stents

Numerous studies have explored the fabrication and performance metrics of vascular stents, utilizing braiding techniques to enhance their structural and mechanical properties.

One study investigated a braided net structure of poly-p-dioxanone (PDO) monofilament with varying braiding densities, undergoing a rigorous 16-week evaluation (Wang and Zhang, 2016). The *in vitro* degradation kinetics of the PDO monofilament were analyzed at monthly intervals, focusing on surface morphology, monofilament crystallinity, mass attrition, flexural rigidity, and tensile attributes. Initial observations indicated that degradation began with changes in the morphological domain, leading to a temporary increase in crystallinity, monofilament flexural rigidity, and the overall mechanical integrity of the stent. In subsequent stages, tensile strength decreased, resulting in brittleness and rigidity. Eventually, the crystalline region degraded, causing a decline in the stent's mechanical properties. Empirical results showed that PDO-crafted intravascular stents with braided architectures maintained appropriate structural integrity for vascular endothelium over 10 weeks.

Further studies examined the influence of guiding yarns, or “runners,” on the mechanical dynamics of poly-l-lactic acid (PLLA) braided stents across axial, circumferential, and helical vectors (Zhao *et al.*, 2021). By braiding PLLA monofilaments over a copper template, a six-pin stent was created, measuring 8 mm in diameter and 20 mm in length, with a helical pitch of 7.74 mm. The mechanical resilience of these stents was significantly influenced by the runners' directional orientations. Notably, runners, especially those in helical trajectories, significantly enhanced the stent's radial tenacity and flexural rigidity compared to controls.

### C. Wearable sensors

Braiding techniques have proven to be highly effective in the development of advanced wearable sensors, enhancing their electromechanical properties and strain detection efficiency.

One study investigated the use of braided composite yarns (BYs) in fabricating wearable strain sensors (Pan *et al.*, 2019). These sensors were synthesized through *in situ* polymerization of polypyrrole (PPy) on the yarn surface, controlled by polydopamine templating (BYs-PDA). The unique braided morphology of the BYs imparted excellent characteristics to the resulting BYs-PDA-PPy strain sensors. They demonstrated good long-term endurance and electrical heating capabilities.

Another study reported the fabrication of a flexible sensor using a braided construction of linear ionogels (IG-BPS) for monitoring human physiological signals and daily movements (Zhang *et al.*, 2023). The braided structure of the sensor disperses stress and forms numerous piezoresistive knots, ensuring high sensitivity and durability. The IG-BPS sensor exhibited great flexibility, a broad pressure application range (0–30 kPa), high sensing behavior (S = 0.137 kPa − 1, GF = 0.029), and stability over more than 1000 cycles. This sensor effectively detects normal movements and subtle epidermal pulse waves at various body locations, including heartbeats and pulse bounding, making it crucial for real-time health monitoring, especially cardiovascular health during intensive exercise. A further study explored the application of a piezoelectric braided cord made from poly-l-lactic acid (PLLA) for sensing vital signs during sleep (Tajitsu *et al.*, 2020). This prototype system consisted of a bed sheet and a pillowcase embroidered with the PLLA braided cord, enabling the observation of body movements and vital signs *in situ*. Preliminary experiments demonstrated the system's ability to measure pulse waves and breathing while the subject lay on their back or side.

Additionally, novel textile temperature sensors based on strain-relieved braiding constructions have been developed, offering significant monitoring possibilities for e-textiles and medical textiles (Wendler *et al.*, 2019). This research focused on the theoretical foundations, manufacturing, and mechanical and thermal testing of these textile-based sensors. The scalable sensor yarns use a helical stainless-steel wire with a median basic resistance of about 0.81 Ω/mm. Within the temperature range of 22–40 °C, the developed sensor yarn showed a mean linearity deviation of 0.028 K.

### D. Importance and limitations of braiding

Braiding is a versatile and widely utilized technique in the fabrication of medical devices, offering unique advantages and facing certain limitations. One of the primary benefits of braiding is its ability to enhance the structural integrity of medical devices (Emonts *et al.*, 2021). This technique creates robust and flexible frameworks essential for applications like vascular grafts and stents, where durability and adaptability to bodily movements are crucial. Braiding has the merit of superior flexibility and robust structural stability (Shukla *et al.*, 2024). The flexibility and conformability of braided structures enable them to fit complex anatomical shapes, ensuring a better fit within the vascular system and reducing the risk of complications. Additionally, the braiding process allows precise control over the material's porosity, which is particularly beneficial in vascular grafts (Guan *et al.*, 2019). This control over permeability helps manage blood flow, prevent clotting, and promote natural tissue integration.

Moreover, braiding enhances the functionality of medical devices by enabling the integration of multiple materials and functionalities within a single device. This capability is especially advantageous in wearable sensors, where embedding conductive fibers can provide electronic functionality while maintaining the necessary comfort and flexibility for the user (Manvi *et al.*, 2019). Thus, braiding not only improves the structural and functional aspects of medical devices but also contributes to their overall effectiveness and usability.

However, the braiding technique is not without its limitations. One of the main challenges is the complex manufacturing process, which requires specialized equipment and expertise (Pedde *et al.*, 2017). This complexity can increase production costs and may limit the widespread adoption of braiding in some medical applications. Additionally, the need for precise control and expertise in braiding can pose challenges in scaling up production for mass manufacturing. Braiding, despite its intrinsically limited porosity and unidimensional configuration, which may impede cellular ingress and subsequent proliferation, still presents distinct material advantages, particularly for load-bearing tissues (Aibibu *et al.*, 2016). Despite these limitations, the benefits of braiding in terms of structural integrity, flexibility, porosity control, and enhanced functionality make it a valuable technique in the development of advanced medical devices.

To summarize, the requisite materials and parameters essential for biomedical end-products, as well as the strengths and limitations intrinsic to each textile modality, are shown in Table I.

**TABLE I.** Tabular representation of the materials and parameters needed for the successful finished product for biomedical applications and the advantages and disadvantages of each textile technique.

Textile technique	Materials	Parameters	Advantages	Disadvantages
Electrospinning	Polymer solution (fiber + solvent), spinneret, syringe, syringe pump, collector	Properties of polymer solution (Mw of polymer, %w/v of solution), distance between the collector and the needle tip, nozzle diameter, environmental condition (ambient temperature and relative humidity), applied voltage	Ability to create multiple fiber assemblies. Most flexible method. Enables adjustment tailored to certain application and great mechanical strength. It has control over porosity, pore size distribution, and scaffold architecture. Large specific area and pore volume	Poor cell infiltration and migration due to the close packing of scaffold fibers, toxicity of the residual solvent, low mechanical strength of scaffolds for load-bearing applications
Electrostatic flocking	Substrate (vascular graft, cardiac patches, stents), adhesives, flock fibers	Fiber density, flock time, Fiber length, applied voltage	Exceptional surface area to volume ratio, efficient cell cultivation, great porosity, strong mechanical characteristic	Stringent electrical conductivity requirement, inability to produce individual flock fibers
Weaving	Warp, weft	Weave used, thread spacing, material used, warp and weft density, yarn fineness, interlacing law, quantity of warps and weft per square inch	Less mechanically demanding than others, higher strength, greater stability than knitted and braided fibers	Manually made, smaller pores and less porous than knitted fabrics, lower flexibility than knitted fabrics
Knitting	Warp, weft	Density of needle (gauge), type and size of yarn, tension applied, knitting technique, stitch pattern	Greater pores, superior softness, elasticity and bursting qualities than weaving and braiding, higher porosity and more favorable to cell growth than woven fabrics.	Not good for cell seedling, challenging to modify in different directions
			Good for creation of load-bearing tissue due to its capacity to stretch	
Braiding	Three or more yarn	Fiber material, braiding angle, fiber density, layer count	Load-bearing capacity, strong tensile strength and mechanical flexibility, damage tolerance and resistance in bending, torsion, and traction, improved abrasion resistance	Minimal porosity

## IX. LIMITATIONS AND FUTURE DIRECTIONS

Textile-based vascular grafts and cardiac patches offer promising alternatives to traditional synthetic and autologous grafts, but several limitations must be addressed to ensure their effectiveness in clinical applications. One key limitation is the potential for loose edges in woven or knitted scaffolds, which can reduce suture retention and mechanical stability during implantation (Miranda *et al.*, 2020). This issue becomes particularly significant in vascular grafts where poor edge retention can lead to graft failure. Additionally, excessive porosity in textile-based scaffolds, although beneficial for cell infiltration and nutrient exchange, can cause unintended problems such as blood leakage and reduced mechanical integrity (Kangazi and Merati, 2023). Managing the balance between porosity and structural integrity is crucial for ensuring that these scaffolds function effectively *in vivo*. Techniques like multi-layered scaffold designs and advanced fiber manipulation methods are being explored to mitigate these issues by controlling pore size and enhancing mechanical strength (Doersam *et al.*, 2022).

Compared to commercially available products such as ePTFE and Dacron, textile-based scaffolds demonstrate superior performance characteristics in terms of anisotropic elasticity, tensile strength, and biocompatibility (Morris and Murray, 2020). These scaffolds better mimic the behavior of native tissues by allowing cellular integration and tissue regeneration, while traditional synthetic grafts often suffer from poor cell adhesion and rigid mechanical properties. However, despite these advantages, textile-based technologies such as weaving, knitting, and electrospinning still face challenges in achieving the long-term durability and mechanical resilience needed for dynamic environments like the cardiovascular system.

Future research should focus on optimizing fiber density and structural layering to improve mechanical properties and suture retention without compromising the scaffold's biocompatibility. Moreover, combining textile techniques with biodegradable coatings and growth factor-infused materials could further enhance the regenerative potential of these scaffolds. Additionally, the development of smart textiles that respond to physiological changes in real time represents a promising direction for improving the functionality of vascular grafts and cardiac patches. Innovations in manufacturing technologies, such as 3D weaving and automated textile production, will also be critical for scaling up these advanced scaffolds for widespread clinical use.

## X. CONCLUSIONS

This comprehensive research surveys the available textile technologies, examining both their traditional and innovative applications. It further examines the etiology, classifications, and therapeutic interventions associated with cardiovascular disease, underscoring both conventional and novel methodologies. The literature corroborates the efficacy and intrinsic benefits of textile modalities in the realm of tissue engineering. Fine-tuning the parameters inherent to these technologies profoundly influences suitability for tissue engineering applications of the resultant scaffolds.

Each textile modality has unique attributes, offering bespoke functionalities for specific biomedical needs. Textile engineering emerges as a robust and effective technology for tissue engineering. Electrospun nanofibers, characterized by small diameters, confined pore dimensions, and expansive surface areas, exhibit the potential to remediate compromised vascular structures. Electrospinning, with its proficiency in generating nanometer-scaled fibrous structures, facilitates the crafting of yarns, which can subsequently be manipulated through weaving, knitting, or braiding to conceive scaffolds. Furthermore, electrostatic flocking augments the attributes of scaffolds derived from various modalities, owing to its pronounced porosity and anisotropic attributes.

Constructs crafted through weaving provide cardiovascular tissue engineering solutions for cardiac patches, vascular grafts, stents, and pacemakers. Pharmacologically augmented scaffolds, infused with agents like heparin, hold promise in addressing vascular anomalies. A knitted polytetrafluoroethylene (PTFE) construct exhibits the potential in ameliorating myocardial infarction, given its durability and robustness. Tubular scaffolds, manifested through knitting high-fineness PLA fibers, stand out for their tensile resilience, rendering them apt for load-bearing applications.

Human mesenchymal stem cells (hMSCs) augment the efficacy of scaffolds for tissue regeneration due to their proclivity to stimulate cellular dynamics and recapitulate the mechanical proprieties akin to authentic tissues. Key parameters, such as braiding angle, braiding density, and the incorporation of a poly(glycerol sebacate) (PGS) coating, significantly influence graft performance. These parameters in turn affect inflammation levels, extracellular matrix genesis, graft dilation, neovascular distensibility, and graft longevity.

Textile-based scaffolds, including those developed through weaving, knitting, and electrospinning, offer several advantages over commercially available synthetic products such as ePTFE or Dacron. These scaffolds demonstrate superior mechanical properties, including anisotropic elasticity and tensile strength, which more closely resemble the behavior of native tissues. Compared to traditional synthetic grafts, textile-based scaffolds provide better cellular integration, porosity, and biocompatibility, facilitating faster tissue regeneration and reducing the risk of thrombosis. This makes them a promising alternative for both cardiac patches and vascular grafts, providing enhanced durability and long-term functionality in dynamic environments such as the cardiovascular system.

Paving the way for future endeavors, researchers should consider amalgamating electrospinning with auxiliary techniques to explore the synergies between electrostatic flocking and constructs derived from electrospinning, weaving, braiding, or knitting for biomedical exigencies. These crafted structures might further be enhanced through seeding with heparin, growth factors, or hMSCs.

## Data Availability

Data sharing is not applicable to this article as no new data were created or analyzed in this study.
